# Repairing gut barrier by traditional Chinese medicine: roles of gut microbiota

**DOI:** 10.3389/fcimb.2024.1389925

**Published:** 2024-07-04

**Authors:** Yaochuan Zhou, Dandan Zhang, Hao Cheng, Jinlu Wu, Juan Liu, Wuwen Feng, Cheng Peng

**Affiliations:** ^1^ State Key Laboratory of Southwestern Chinese Medicine Resources, School of Pharmacy and School of Basic Medical Sciences, Chengdu University of Traditional Chinese Medicine, Chengdu, China; ^2^ TCM Regulating Metabolic Diseases Key Laboratory of Sichuan Province, Hospital of Chengdu University of Traditional Chinese Medicine, Chengdu, China; ^3^ Key Laboratory of the Ministry of Education for Standardization of Chinese Medicine, Chengdu University of Traditional Chinese Medicine, Chengdu, China

**Keywords:** gut barrier, gut microbiota, gut microbiota metabolite, traditional Chinese medicine, intestinal tract integrity

## Abstract

Gut barrier is not only part of the digestive organ but also an important immunological organ for the hosts. The disruption of gut barrier can lead to various diseases such as obesity and colitis. In recent years, traditional Chinese medicine (TCM) has gained much attention for its rich clinical experiences enriched in thousands of years. After orally taken, TCM can interplay with gut microbiota. On one hand, TCM can modulate the composition and function of gut microbiota. On the other hand, gut microbiota can transform TCM compounds. The gut microbiota metabolites produced during the actions of these interplays exert noticeable pharmacological effects on the host especially gut barrier. Recently, a large number of studies have investigated the repairing and fortifying effects of TCM on gut barriers from the perspective of gut microbiota and its metabolites. However, no review has summarized the mechanism behand this beneficiary effects of TCM. In this review, we first briefly introduce the unique structure and specific function of gut barrier. Then, we summarize the interactions and relationship amidst gut microbiota, gut microbiota metabolites and TCM. Further, we summarize the regulative effects and mechanisms of TCM on gut barrier including physical barrier, chemical barrier, immunological barrier, and microbial barrier. At last, we discuss the effects of TCM on diseases that are associated gut barrier destruction such as ulcerative colitis and type 2 diabetes. Our review can provide insights into TCM, gut barrier and gut microbiota.

## 1 Introduction

Gut barriers are essential defenses in the intestinal tract that provide robust protection against harmful microorganisms, both internal and external. These barriers include physical, chemical, immunological, and microbial components, each with its own distinct structure and function. Dysfunction of the gut barrier has been implicated in various diseases such as irritable bowel syndrome, celiac disease, enteric infections, nonalcoholic fatty liver disease (NAFLD), type 2 diabetes (T2D), insulin resistance (IR), and inflammatory bowel disease (Crohn’s disease and ulcerative colitis) (Turner, 2009; König et al., 2016). Because of the importance of gut barrier in the development of diseases, gut barrier has played increasingly important role in understanding the development of diseases and developing drugs for disease treatment.

Gut microbiota, the largest micro-ecological system in the human body, plays a crucial role in maintaining the normal function of the host (Ma et al., 2019). The gut microbiota is composed of over 1500 species, including bacteria, viruses and other microorganisms, with certain phyla such as *Firmicutes*, *Bacteroides*, *Actinobacteria*, *Proteobacteria*, and *Verrucomicrobia* being particularly important (Fava et al., 2019). The composition of the gut microbiota is closely associated with the function of gut barriers and the development of various diseases. A healthy gut microbiota suppresses the growth and translocation of pathogens, strengthens the gut mucosal barrier, and reduces the absorption of toxins (Paolella et al., 2014). It also supports the immune system by enhancing the secretion of antimicrobial factors (Mills et al., 2019). On the contrary, gut microbiota dysbiosis can lead to the development of diseases such as hypertension, gastrointestinal disease, cardiovascular disease, metabolic disease, anxiety, depression and cancer (Afzaal et al., 2022). Consequently, the study of gut microbiota has become an important area of research for understanding disease development and treatment strategies.

In addition to gut microbiota, its metabolites such as short-chain fatty acids (SCFAs), bile acids (BAs), and tryptophan (Trp), also play a crucial role in influencing gut barriers. These metabolites are involved in maintaining the normal function of hosts and the development of diseases. For example, SCFAs are viewed as crucial energy sources and anti-pathogen factors for intestinal epithelial cells (Afzaal et al., 2022). Other than that, SCFAs can upregulate the expressions of zonula occludens 1 (ZO-1), occludin and claudin, which are beneficial for reinforcement of gut physical barrier (Ghosh et al., 2021). Besides, BAs can regulate gut microbial barrier via inducing the transcription of antimicrobial agents such as inducible nitric oxide synthase and interleukin (IL)-18 (Inagaki et al., 2006). Moreover, Trp and its metabolites have been found to be beneficial for strengthening intestinal barrier function by upregulating the expression of the gene concerning mucosal barrier and mucin production (Bansal et al., 2010). Hence, given the importance of gut microbiota metabolites, numerous studies have directed attention to both gut microbiota and its metabolites simultaneously.

In recent years, several evidence has revealed that TCM can directly regulate the structure of gut microbiota and the production of its metabolites, then exerting therapeutic influences (Lyu et al., 2017; Xia et al., 2022). For example, hydroxysafflor yellow A has been demonstrated to alleviate obesity through modulation of the gut microbiota and itsmetabolites. Specifically, supplementation with hydroxysafflor yellow A was found to increase the abundances of *Akkermansia, Butyricimonas*, *Alloprevotella*, and *Romboutsia*, while decreasing the levels of *Lachnospiraceae*. Concurrently, hydroxysafflor yellow A promoted the production of acetate, propionate, and butyrate (Liu et al., 2018a). TCM-gut microbiota interactions also play a role in repairing and strengthening the gut barrier. Qiwei Baizhu Decoction can increase butyrate levels, aiding in the repair of the gut chemical barrier by enhancing mucin 2, a core component of gut chemical barrier (Etienne-Mesmin et al., 2019). Similarly, the extract of Paeoniae Radix Alba promoted *Akkermansia* levels in DSS-induced colitis mice, benefiting the gut chemical barrier in colitis (Yan et al., 2022). Thus, studying the gut microbiota and its metabolites is crucial for understanding how TCM impacts the gut barrier and produces therapeutic effects.

Currently, there is a lack of review explaining how the gut barrier can be regulated through the interfaces amidst gut microbiota, its metabolites and TCM. In this review, we discuss the composition and function of gut barrier, and then the interactions amidst gut microbiota, its metabolites and TCM. Then, we unravel how TCM exert protective and therapeutic influences on gut barrier via modulating gut microbiota and its metabolites. We also discuss the role of TCM in some common diseases with destroyed gut barrier via regulating gut microbiota and its metabolites. Our review can provide insights into TCM, gut barrier and gut microbiota, enabling the development of novel strategy to treat diseases by modulating gut barrier.

## 2 Gut barrier

**Figure 1:**
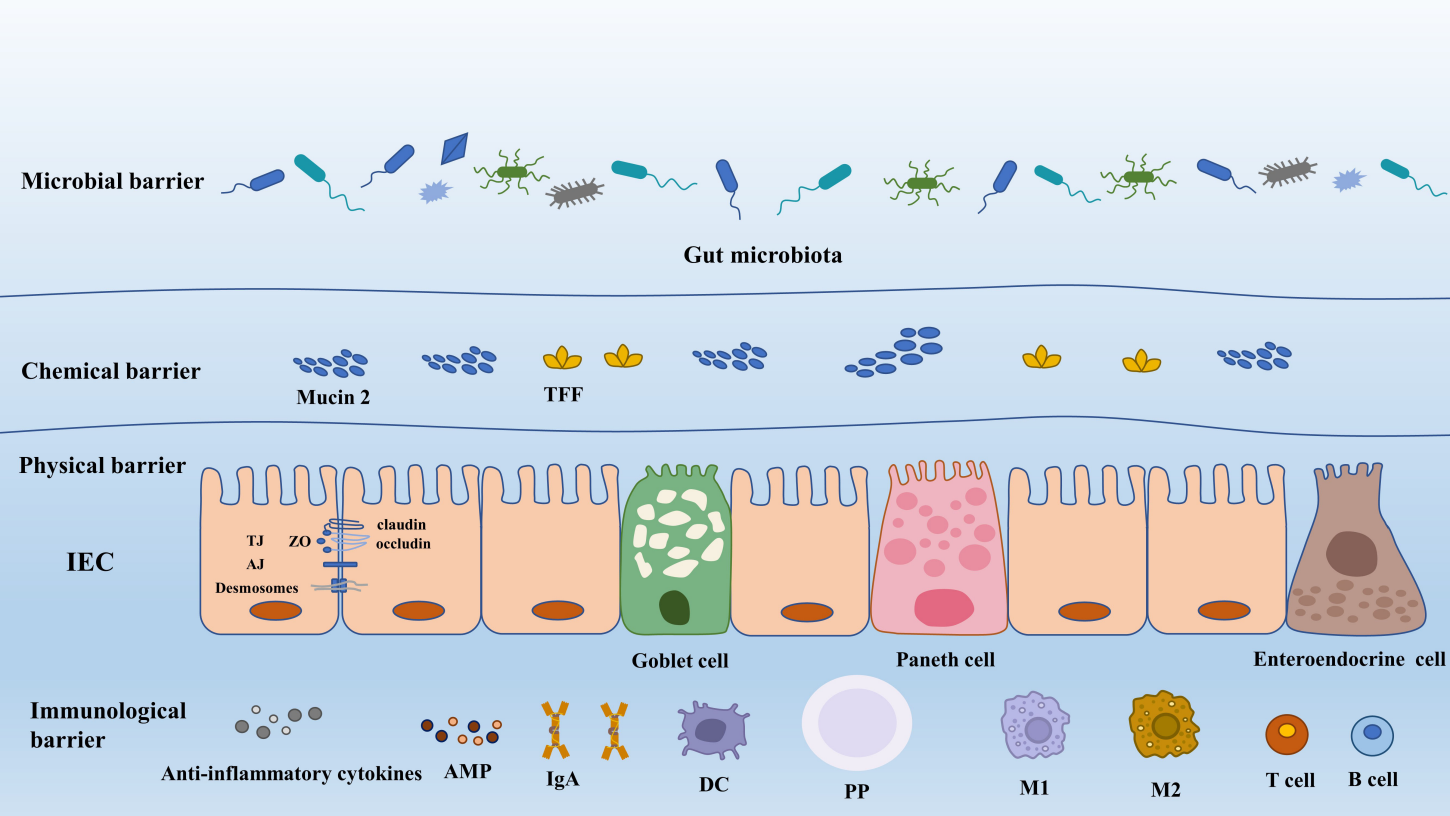
The composition of gut barrier. AJ, adherens junction; AMP, antimicrobial peptide; DC, dendritic cell; IEC, intestinal epithelial cell; IgA, immunoglobulin A; M1, macrophage 1; M2 macrophage 2; PP, Peyer’s patch; TFF, trefoil factor; TJ, tight junction; ZO, zonula occludens.

The gut barrier is a sophisticated system providing two important functions for the survival of the individual. Firstly, it permits nutrient absorption and secondly, it protects the body from the invasion of deleterious microorganisms. Generally, gut barriers comprise four aspects, i.e., physical barrier, chemical barrier, immunological barrier, and microbial barrier (**Figure 1**).

### 2.1 Gut physical barrier

The gut physical barrier is composed of intestinal epithelial cells (IECs) and the tight junctions between them. It is composed of a single layer of epithelial cells that are interspersed with specialized differentiated cells, including six distinct cell lineages. Enterocytes, comprising 80–90% of all differentiated cells, are highly efficient in absorbing nutrients and water. Microfold cells (M cells) play a crucial role in sampling the contents of the gut and transporting luminal antigens to the underlying immune cells within the Peyer’s patches (PPs), thus regulating immune responses. Goblet cells are responsible for secreting mucus, which serves to lubricate and protect the intestinal surface against bacteria. Paneth cells produce antimicrobial compounds and provide niche factors for the growth and maintenance of intestinal stem cells. Chemosensory tuft cells mediate immune responses, while enteroendocrine cells produce hormones involved in regulating appetite and insulin release (Beumer and Clevers, 2021). Paneth cells and microfold cells are exclusively present in the small intestine, whereas enterocytes, goblet cells, enteroendocrine cells, and tuft cells are found in both small intestine and colon. These cells form a continuous and polarized layer, effectively acting as the boundary between luminal content and lamina propria (Ghosh et al., 2021).

Gut physical barrier can prevent the luminal content (endotoxin, viruses, food antigens, and toxins) and the invading pathogens from intruding into the underlying tissues and producing adverse effects on the host. However, the barrier formed by IECs can be selectively permeable to substances beneficial to the host, such as sugars, water, amino acids, electrolyte, SCFAs, and other gut microbial metabolites. The robust defensive function of IECs and their maintenance of cellular integrity rely on their architectural advantage: tight junction (TJ), adherence junction, gap junction and desmosomes. Besides, these structures make contributions to directing intracellular transportation and functional substances secretion (antimicrobial peptide (AMP), mucin protein), and more significantly, mediating organelle movements (Natividad and Verdu, 2013).

TJ can effectively modify the ability of IECs to regulate the degree of adhesion between cells, the paracellular and transcellular permeability, as well as gut barrier health (Chiba et al., 2008). TJ structure is composed of transmembrane proteins. They are claudin, occludin, tricellulin and junctional adhesion molecules (JAMs) and intracellular plaque proteins such as zonula occluudens (ZO) and cingulin (Suzuki, 2020). Claudins, a diverse protein family consisting of at least 27 members, exhibit various functions in cellular barriers. Some claudins (claudin-3,4,7 and 14) (Van Itallie and Anderson, 2006) act as barriers for macromolecules and ions, while others, like claudin-2, possess properties that form pores facilitating the passage of ions and water (Lu et al., 2013). Besides, claudin-1 can help to modulate epithelial homeostasis by the regulation of notch-signaling and more importantly it can enhance epithelial proliferation in a notch-dependent manner (Pope et al., 2014).

Occludin, associated with cytosolic plaque proteins, plays a role in providing a scaffold for TJ. The scaffold molecules involved in TJ formation include ZO-1 and cingulin. ZO-1, in particular, is essential for coordinating TJ assembly and cell polarization by connecting transmembrane TJ proteins (such as claudin and JAMs) with components of the cytoskeleton (Ikenouchi et al., 2007). In addition to the role in TJ formation, ZO-1 is associated with epithelial proliferation, repair, and survival. For example, epithelial cells lacking ZO-1 exhibited significant impairments in their ability to efficiently initiate proliferation during wound healing. This impairment is attributed to defects in WNT signaling and the orientation of the mitotic spindle (Kuo et al., 2022).

### 2.2 Gut chemical barrier

The mucus layer in the intestinal tract acts as a protective barrier, attaching to the surface of intestinal epithelial cells and safeguarding them against mechanical, chemical, and biological attacks. This mucus layer functions as a chemical barrier and is composed of multiple proteins (mucin 2, chloride channel accessory-1, Fc fragment of IgG binding protein, and zymogen granule protein 16), lipids, ions, and water (constituting approximately 95%) (Suriano et al., 2022). The core of the mucus layer is mucus protein, also called mucin, which forms a tight and protective coating over the intestinal tissue, providing lubrication and safeguarding the cells (Paone and Cani, 2020). The composition and thickness of the mucus layer vary throughout the intestinal tract, giving rise to different physiological functions. In the small intestine, the mucus layer is a single layer characterized by the presence of antimicrobial peptides immunoglobulin A (IgA) and endogenous enzyme inhibitors. It is described as a loosely organized but efficient barrier for epithelial cells (Suriano et al., 2022). In contrast, the mucus layer in the large intestine consists of two distinct layers: the inner and the outer layer (Johansson et al., 2011). The inner mucus layer undergoes continuous replenishment through the production of mucin 2. It is firmly anchored to goblet cells and maintains its attachment to the epithelium, ensuring a persistent barrier. In mice, the inner mucus layer has a pore size 0.5 μm, making it impermeable to bacteria. However, endogenous proteases can convert the inner mucus into the outer mucus layer (Birchenough et al., 2015; Hansson, 2019). The outer mucus layer has a loose and net-like structure with larger pores, allowing for the permeability of bacteria or beads up to 0.5 μm (Paone and Cani, 2020). The mucus layer in the large intestine plays a dual role: it prevents direct contact between luminal bacteria and epithelial cells, while also providing a habitat for commensal bacteria through its favorable ecological niche (Suriano et al., 2022).

The primary component of mucus is the mucin 2, a glycoprotein consisting of approximately 5,200 amino acids and containing around 80% glycans, predominantly O-glycans. These highly glycosylated mucins possess multiple O-glycan epitopes that serve as attachment points for commensal bacteria. Through the action of glycan enzymes, commensal bacteria can cleave and utilize the O-glycans present in mucin as an energy source while preserving the overall structure of mucin 2 (Luis and Hansson, 2023). The biosynthesis, glycosylation, storage, and secretion of mucins are carried out by goblet cells. After secretion, the packed mucins require exposure to increased pH and reduced calcium concentration in order to properly expand. This process involves the participation of bicarbonate ions, which are provided by the cystic fibrosis transmembrane conductance regulator channel. The expansion of the packed mucins results in the formation of a net-like structure that can expand in volume by 100–1000 times, effectively binding water and creating a protective barrier (Paone and Cani, 2020). Mucin 2 is found in both the small and large intestine, where it forms the framework of the mucus layer. Besides, mucin 2 is involved in the clearance of outside pathogens. After being infected with *Citrobacter rodentium*, a murine pathogen associated with diarrheagenic A/E *E. coli*, mice lacking mucin 2 (Muc2^−/−)^ showed more severe symptoms compared to wild-type mice. They experienced colon ulceration, overt pathogen and commensal translocation into colonic mucosa and had a high mortality rate of up to 90% (Bergstrom et al., 2010).

The mucus barrier’s ability to operate depends on the specific relationship between the gut microbiota and the mucus layer. In Germ-free rats, it has been observed that the absence of bacteria leads to a thinner or even absent mucus layer in the colon (Szentkuti et al., 1990). Additionally, compared to mice raised conventionally, Germ-free mice were found to have a more permeable inner colonic mucus layer with a lower relative abundance of mucin 2 (Johansson and Hansson, 2012). This highlights the role of bacteria in ensuring the integrity of the mucus barrier. Furthermore, the mucus layer serves as an alternative nutrient source for the gut microbiota, supporting their colonization and persistence in the gut (Schroeder, 2019), which emphasizes the importance of the mucus layer in providing nourishment to the microbiota. In summary, maintaining the integrity of the mucus barrier and promoting the development and survival of the microbiota depend on the interaction between the gut microbiota and the mucus layer. The mucus layer’s health and this interaction are also influenced by the diet. Consuming more fiber encourages the production of gut microbiota metabolites, such as SCFAs (Suriano et al., 2022). Particularly, butyrate can improve the function of the mucus barrier by regulating mucin 2 production and secretion (Etienne-Mesmin et al., 2019). However, diets low in fiber, such as Western diets and high-fat diets, can disrupt the composition of the gut microbiota, reducing its diversity. This disruption weakens the mucus barrier and disturbs immune balance (Statovci et al., 2017; Birchenough et al., 2019). In the absence of dietary fiber, the gut microbiota adapts by relying on host-glycans from mucins as an energy source (Sonnenburg and Sonnenburg, 2014; Earle et al., 2015). Unfortunately, this alteration negatively impacts the integrity of the mucus barrier, may causing it to break down. As a result, the gut becomes more vulnerable to gastrointestinal pathogen infections.

### 2.3 Gut immunological barrier

Gut immunological barrier is a complicated mucosal immune system, primarily comprising three lymphoid structures: Peyer’s patches, the lamina propria and the intestinal epithelia cells (Richards et al., 2016). Immune cells, including Paneth cells, intestinal B cells, T cells, and enterocytes, possess the remarkable ability to secrete bactericidal substances (Lehrer, 2004; Tezuka and Ohteki, 2019). These substances, such as sIgA, lysozyme, *α*-defensin, *β*-defensin, and C-type lectins (such as Reg3-*γ* and Reg3-*β*) (Mukherjee et al., 2009), serve as the first layer of gut immune barrier, contributing significantly to the overall defense mechanisms in the gut. sIgA, a widely distributed antibody, serves dual functions in response to pathogens: pathogen elimination and induction of tolerance. It can eliminate pathogens through immune exclusion, providing nonspecific immunity. Additionally, sIgA possesses neutralizing properties, directly impacting bacterial viability or altering pathogenicity (Li et al., 2020b). For instance, the toxins such as *Clostridium difficile* toxin A and enteropathogenic *E. coli* intimin can both be neutralized by galactose residues of sIgA (Perrier et al., 2006). Furthermore, sIgA can regulate T cells by binding to specific receptors, suggesting its potential role in immunoregulation (Diana et al., 2013). *α*-defensin is the most abundant antimicrobial peptide in the human intestine which can directly kill Gram-negative and Gram-positive bacteria (Bevins and Salzman, 2011). Besides, human *α* defensin 5 (HD5) can neutralize bacterial endotoxin and exert an important influence on regulating host antimicrobial activities (Bevins and Salzman, 2011). Furthermore, the damaged expression of *α*-defensin has been revealed to be correlated with disruption of the intestinal homoeostasis, such as increased intestinal permeability, mucosal damage, inflammation and endotoxin, and HD5 treatment can prevent these injuries through suppressing the proinflammatory cytokines such as IL-1*β*, tumor necrosis factor (TNF)-*α*, IL-6 and chemokines such as CCL5 and MCP1 (Shukla et al., 2018). The similar function has also been exhibited by C-type lectins and Lysozyme, which have been broadly reviewed (Ganz et al., 2003; Bevins and Salzman, 2011; Vaishnava et al., 2011).

Functioning as the secondary layer of the gut immune barrier, intestinal epithelial cells can directly partake in immune monitoring of the gut. Beyond offering direct defense against opportunistic pathogens, these epithelial cells are also involved in transmitting signals to the mucosal immune system by producing cytokines and chemokines (Kuhn et al., 2014). Located in epithelial cell layer, both innate lymphoid cells and intraepithelial lymphocytes (IELs) can produce cytokines that protect the host. For example, upon activation, IELs will express cytokines like IFN-*γ* and keratinocyte growth factor, which act to defend the epithelial cells against injury (Shi et al., 2017). Dendritic cells (DCs) possess the ability to collect mucosal antigens by extending their dendrites and transporting them across the barrier to the mucosal-associated lymphoid tissue. Additionally, DCs can directly eliminate pathogens such as *Salmonella* and *E. coli* by opening the tight junctions and penetrating into the lumen to phagocytose them (Rescigno et al., 2001). In the gut immunological barrier, other immune cells such as regulatory T cells (Tregs) and T helper (Th) cells are also important. These cells help control inflammation and immunological tolerance in addition to aiding in elimination of pathogens (Shi et al., 2017).

The lamina propria is located in the lower layer of intestinal epithelial cells, and it is the third component of gut immune barrier, fulfilling a crucial role in the initial priming and differentiation of adaptive immune cells. PPs are a key constituent of the lamina propria, with their luminal surface covered by a specialized follicle-associated epithelium (FAE) that includes M cells (Owen and Jones, 1974). In mice, these M cells transport luminal antigens, such as those from bacteria and viruses, into the PPs to support optimal IgA responses (Mörbe et al., 2021). PPs contain mature B cells, and the B cell follicles are covered by the subepithelial dome, which is located under the follicle-associated FAE and contains DCs. B cells and T cells get activated when DCs presenting antigen, which starts an immune response (Li et al., 2020b). The activated B cells in the PPs continuously promote the production of plasma cells, which secrete IgA to protect the intestinal tract (Bemark et al., 2012). Concurrently, T cells can rapidly mount an immune response to stimuli from the intestinal tract, producing IL-17 and IL-22 to modulate inflammation (Muñoz et al., 2009).

The microenvironment created by the gut microbiota and its products is a crucial factor that influences the immunity of the intestinal region. Early in life, the process of beneficial microbes colonizing and growing in mucosal tissues is important. It can actively boost and train the host’s immune system in addition to decreasing the incidence of gastrointestinal infections (Weström et al., 2020). For instance, certain bacterial proteins in the mother’s gut during pregnancy, like metabolites of *Escherichia coli* HA107, can trigger antibody production in the mother’s bloodstream. These antibodies can cross the placenta and impact the immune system of the newborn, promoting the production of fetal group 3 innate lymphoid cells (ILC3) and F4/80+ CD11c+ mononuclear cells, which are crucial for early postpartum innate immunity (Gomez de Agüero et al., 2016). Another example is the segmented filamentous bacterium, a commensal bacterium that has been shown to induce and stimulate various types of intestinal lymphoid tissues, leading to the production of sIgA (Lécuyer et al., 2014). Importantly, the immune system also plays a role in maintaining the balance of the gut microbiota. For example, mice lacking matrix metalloproteinase 7 (MMP7) have reduced levels of *α*-defensin, while mice with the human HD5 gene have increased levels. This imbalance affects the composition of bacteria in their intestines, with MMP7-deficient mice having fewer *Bacteroidetes* and more *Firmicutes*, and HD5 mice showing the opposite pattern. However, the total amount of bacteria remains unchanged in both types of mice, underscoring the vital role of *α*-defensin in maintaining microbiota balance (Salzman et al., 2010).

### 2.4 Gut microbial barrier

The narrow gut microbial barrier solely consists of bacteria, whilst the generalized one is principally composed of bacteria, viruses, helminths, bacteriophages, fungi, and archaea (Garcia-Bonete et al., 2023). The main focus of this discussion revolves around bacteria within the gut microbiota. They have been extensively studied due to their significant representation (>90%) among the microorganisms in the gut, and their ease of culture and isolation adds to their research accessibility (Garcia-Bonete et al., 2023). Firstly, the adhesion, chimerism, and binding of microorganisms to intestinal mucosa comprise the specific structure of the gut microbial barrier, which can effectively defend against extraneous pathogens through competing for the mucosal colonization sites and nutrient intake (Hynönen and Palva, 2013). Secondly, gut microorganisms show an evident colonization distribution. Viewed as the most important beneficial bacteria in the gut (Turroni et al., 2009), *Bifidobacterium* and *Lactobacillus* exhibit long-term gut colonization potential (Xiao et al., 2020). They make up the intestinal membranous microbial community, which can be stirred with mucus, then producing protective effect. Besides, the gut microbial barrier is capable of inhibiting intestinal pathogenic bacteria from overgrowing and translocating through promoting epithelial cell proliferation and differentiation, and activating intestinal mucosal immunity (Purchiaroni et al., 2013), by which severe inflammation responses and oxidative stress can be greatly relieved. Moreover, the bacterial surface layer protein or S layer protein are important to the ability to adhere, aggregate, and regulate T cell immune response and antigen variation, which also impact gut immune function. *Lactobacillus* surface S protein layer can adhere to the host epithelial cell, and competitively suppress the colonization and invasion of pathogenic microorganisms (Hynönen and Palva, 2013).

It has been unraveled that host mucosal binding protein can be found in hairs of *Lactobacillus rhamnosus GG*, which may be connected to competitive colonization of mucosa sites (Kankainen et al., 2009). *Bifidobacterium bifidum* has been proved to greatly suppress the attachment and intrusion of *salmonella* and *E.coli* O157:H7 by regulating relative virulence factors (Bayoumi and Griffiths, 2012). Additionally, probiotics can produce organic acid lowering intestinal pH and Eh, assisting to suppress the growth of pathogens and excluding harmful substances (Vollaard and Clasener, 1994). When in intestinal homeostasis or active intestinal inflammation, hypoxia and hypoxia-inducing factors dominate the regulation of normal intestinal metabolism and barrier function (Glover et al., 2016). The consumption of oxygen by probiotics in the gut contributes to maintaining an oxygen-free environment and affecting the growth of bacteria, then regulating the ecological balance of gut microbiota (Glover et al., 2016). Furthermore, studies have shown that *Lacotobacillus acidophilus* was able to adhere to colorectal cancer-associated cells HT-29 and Caco-2, promoting the expression of ZO-1 and phosphorylation of claudin and occludin, enhancing the assembly of TJ then consolidating gut physical barrier (Montalto et al., 2004; Ashida et al., 2011). Moreover, the protective function of the gut chemical barrier can be indirectly achieved because gut microbiota composition can affect the gut mucosal barrier function, which has been demonstrated by a study (Jakobsson et al., 2015) where two genetically identical mouse colonies, respectively raised into two different rooms with the same facilities, showed discrepancies in gut mucus properties and gut microbiota strains. Interestingly, this caused the ensuing fact that their ilka defensive function of mucus layers differed a lot. Besides, the probiotic community also plays a crucial role in regulating intestinal immunity. the function of sole specific bacteria has been reported such as *Bifidobacterium longum BL-10*, which can exhibit great immune protective functionality by modulating inflammatory cytokines and TLR4/NF-*κ*B signaling pathways, through which the gut microbiota disorders and immunological injuries caused by lipopolysaccharide (LPS) in mice can be greatly restored (Dong et al., 2022). Moreover, probiotics can exert a modulatory influence on intestinal macrophages and DCs, leading to the altered secretion of anti-inflammatory cytokines, such as IL-10, IL-12, and TGF-*β*. Furthermore, probiotics have the capacity to regulate the balance between Th17/Tregs and Th1/Th2 cell subsets, as well as promote the levels of immunoglobulins in the intestinal fluid. Collectively, these probiotic-mediated mechanisms serve to boost the overall intestinal immune response (Purchiaroni et al., 2013).

## 3 Interactions between gut microbiota and TCM

**Figure 2:**
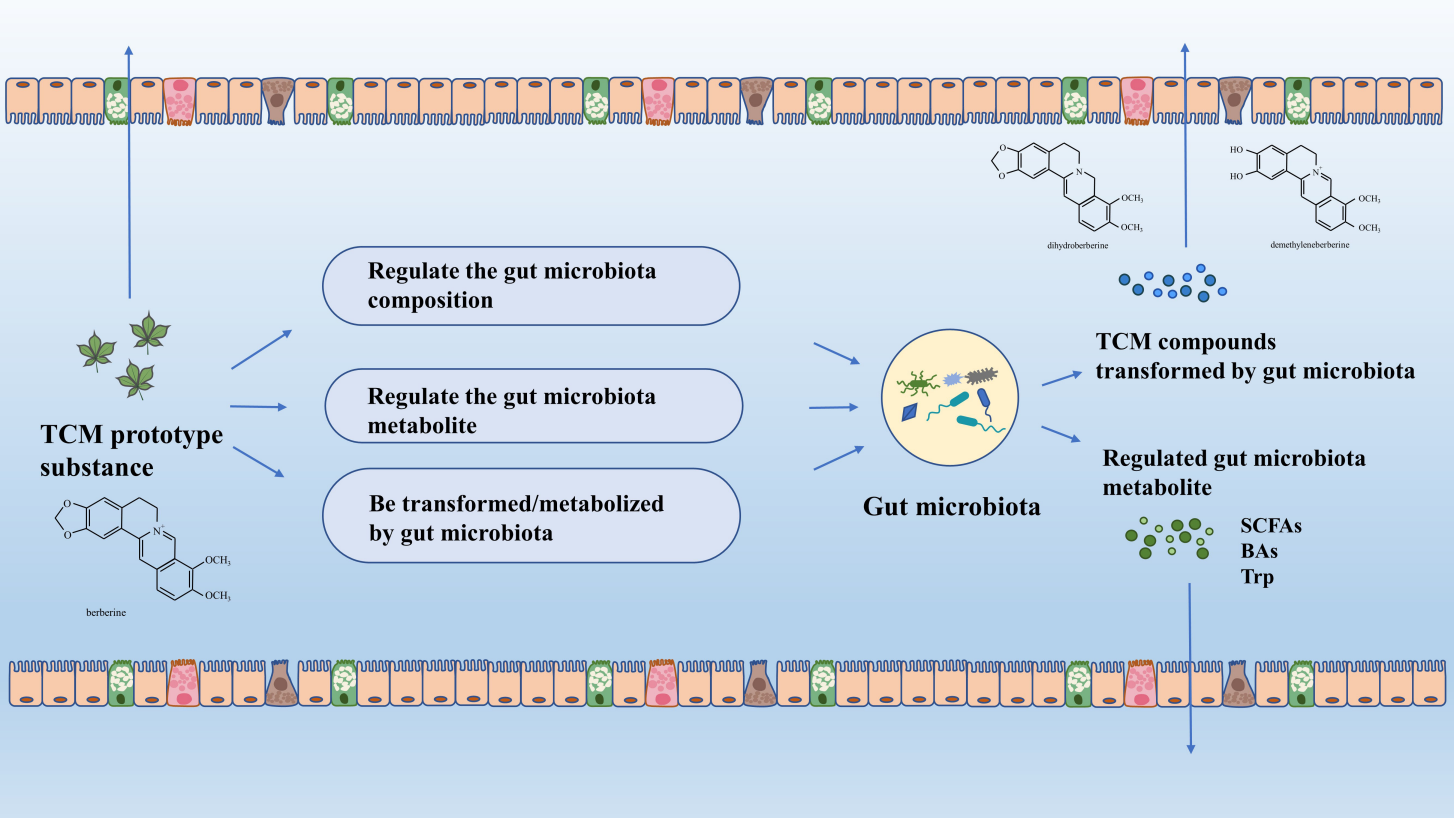
Interactions between gut microbiota and TCM. BAs, bile acids; SCFAs, short chain fatty acids; TCM, Traditional Chinese Medicine; Trp, tryptophan.

In recent years, the interactions between gut microbiota and TCM has been extensively studied to explore the mechanisms of how diseases can be ameliorated by TCM compounds. In the whole process of transmission in gastrointestinal tract, some TCM molecules will be absorbed in the small intestine, which can act on the immune system by promoting the production and secretion of antimicrobial enzymes and antimicrobial substance to avoid colonization of extraneous and indigenous pathogens (Bevins and Salzman, 2011). Other TCM compounds that are hard to absorb will reach the colon and interact with gut microbiota, the processes of which mainly include: (1) TCM can be metabolized into other type of metabolites with better bioavailability and stronger therapeutic effects on the host, (2) TCM can directly and noticeably alter the composition of gut microbiota community, (3) TCM can modulate the gut microbiota metabolites (**Figure 2**).

### 3.1 Gut microbiota transform TCM compounds

After orally taken-in, TCM will not be completely absorbed especially those with low oral bioavailability such as polyphenols, flavonoid, polysaccharide, and saponins (Yue et al., 2017). Thus, they can encounter gut bacteria and interact with each other once arriving at the colon, and these undigested compounds can be transformed into smaller molecules by microbial fermentation. These reactions include hydrolysis, oxidation, decarboxylation, reduction, isomerization, demethylation, deamination, and others (Wilson and Nicholson, 2017). For example, most complex polysaccharides can be translated into SCFAs under the action of carbohydrate enzymes secreted by gut microbiota (El Kaoutari et al., 2013). Another example is polyphenols, which are characterized by low bioavailability. The phenols are converted into molecules with low molecular weight and higher bioavailability, thus they can be absorbed more easily by the host (Aura, 2008). Intriguingly, these metabolites can in turn change the composition of microbiota in a beneficial way (Filosa et al., 2018). To accomplish the reactions above, a battery of enzymes with excellent ability to metabolically activate or inactivate drugs are necessary, such as *β*-glucuronidases, *β*-glucosidase, dioxygenase, CoA ligase (Currò, 2018). What is worth noting is that the reactions between TCM compounds and gut microbiota may not be achieved in one step but in succession. To take flavonoid glycosides as an example, they are transformed by two steps: de-glycosylation and de-methylation, and then the ensuing skeleton fission (Selma et al., 2009). Some of the gut microbiota-transformed TCM compounds can undergo enterohepatic circulation, in which these compounds will be irreversibly combined with glucuronic acid by hepatic enzymes such as UDP-glucuronosyltransferase, and then be released into duodenum. Once reaching the intestine, *β*-glucuronidase from microbiota will disassemble these conjugates, which means the prototype TCM compounds could be produced again and then be utilized by the host repeatedly (Feng et al., 2019). Baicalin can be a standard representative of the compounds discussed above for its performance in enterohepatic circulation (Xing et al., 2005).

### 3.2 TCM modulate the composition of gut microbiota

TCM has four types of effects on gut microbiota: promotion, inhibition, elimination, and new colonization. Promotion, inhibition, and elimination directly impact gut microbiota abundance, while indirect effects can include all four types (Feng et al., 2019). Through these mechanisms, TCM selectively helps to preserve the balance of gut microbiota composition in the face of pathogen invasion, thereby preventing the establishment of exogenous or endogenous pathogens in both healthy and diseased states. There are numerous TCM with the function of promotion and inhibition (**Tables 1**, **2**). For example, the abundance of the genera *Enterorhabdus*, *Odoribacter*, *Ruminococcaceae_UCG_014*, *Ruminococcaceae_UCG_010*, *Enterococcus* and *Ruminiclostridium_5* showed a noticeable increase after treatment of glycyrrhiza polysaccharide (GCP) in mice intestine, and this change probably revealed the evidence supporting the anti-tumor function of GCP (Zhang et al., 2018a). Cinnamon essential oil can inhibit *Escherichia coli* and *Staphylococcus* by directly interacting with the targeted bacteria (Zhang et al., 2016). Besides, researches examining the elimination of gut microbiota have revealed that groups receiving treatment with TCM showed the presence of specific operational taxonomic units (OTUs) that were absent in the model group. Conversely, the model or normal group contained OTUs that were not detected in the TCM-treated group (Xu et al., 2013; Yang et al., 2017).

**Table 1:** Therapeutic efficacy of TCM complex extract on gut barrier, gut microbiota, and gut microbiota metabolites.

TCM	Disease	Improved barrier and corresponding altered indicators	Variation of gut microbiota	Variation of gut microbial metabolites	Reference
Fufang Zhenzhu Tiaozhi Capsule	NASH NAFLD	Physical barrier: claudin-2, claudin-4, ZO-1, E-cadherin, occludin ↑	*Bacteroidia*, *Verrucomicrobiae*, *Epsilonproteobacteria*, *Clostridia* ↓	SCFA ↑ BA: CDCA, UDCA, NorDCA, DCA ↑ Fatty acids ↑	(Lan et al., 2022)
Pien Tze Huang	NASH	Physical barrier: E-cadherin ↑	*Lactobacillus* (e.g., *L.acidophilus, Lactobacillus plantarum* (*L. plantarum*)), *Lactococcus (e.g., Lactococcus lactis)*, *Bacillus* (e.g., *Bacillus subtilis*) ↑ *Citrobacter*, *Pseudomonas ↓*	BA: TUDCA, TCA, TDCA, GCA ↑	(Zeng et al., 2024)
Shugan Xiaozhi Decoction	NAFLD	Physical barrier: ZO-1, occludin ↑ Immune barrier: regulation of sIgA TNF-*α*, IL-1*β*, MCP-1, TGF-*β*1 ↓	*f_Prevotellaceae* ↑ *f_Desulfovibrionaceae*, *f_Erysi-Pelotrichaceae* ↓	–	(Yang et al., 2022a)
Qiwei Baizhu Decoction	Diarrhea	Chemical barrier: mucin 2 ↑	*Verrucomicrobia*, *Akkermansia* ↑ *Turicibacter*, *Flavonifractor* ↓	SCFA: acetate, propionate ↑ butyrate↓	(Sun et al., 2021b)
Flos Abelmoschus Manihot	DSS-induced colitis	Physical barrier: occluding, CDH1, TJP1, ZO-1 ↑, Chemical barrier: mucin 2 ↑ Immune barrier: restore TH17/Treg balance IL-17, IL-22, IL-23 ↓ IL-10 TGF-*β* ↑	Phylum level: *Firmucutes* ↑, *Bacteroidetes* ↓, Genus level: *Lachnospiraceae, Alistipes, Lactobacillus, Bilophila, Desulfovibrio *↑	SCFA: acetate, butyrate ↑	(Zhang et al., 2019)
Qingchang Huashi Formula	DSS-induced colitis	Physical barrier: ZO-1, occludin ↑ Chemical barrier:mucin 2 ↑ Immune barrier: reg3-*γ* ↑	*Firmicutes* ↑ *Bacteroidetes* ↓	SCFA: butyrate, isobutyric acid (ISO-BUT), valeric acid ↑, acetic acid, 4-methylisovaleric acid (4-MeVA) ↓ BA: CA, T-CA, T-*β*-MCA ↓, DCA, UDCA, *β*-MCA, T-DCA, T-UDCA ↑	(Hu et al., 2021)
Rhubarb Peony Decoction	UC	Immune barrier: regulate TH17/Treg balance IFN-*γ*, IL-6, TNF-*α*, IL-17A, IL-22 ↑, TGF-*β* ↓	*Firmicutes*, *Actinobacteria*, *Butyricicoccus pullicaecorum* ↑ *Proteobacteria*, *Bacteroidetes* ↓	SCFA: acetate, propionate, butyrate ↑	(Luo et al., 2019)
Qingchang Wenzhong Decoction	UC	Physical barrier: ZO-1, E-cadherin, Occludin ↑, Chemical barrier: mucin 1, mucin 2, goblet cells ↑	*Lactobacillus ↑* *Bacteroides_vulgatus, Bacteroides_uniformis ↓*	Trp: 5-Methoxyindoleacetate, 5-Hydroxyindoleacetylglycine, 1H-Indole-3 acetamide ↓	(Sun et al., 2021c)
Aqueous extract of Paeoniae Radix Alba (*Paeonia lactiflora* Pall.)	DSS-induced colitis	Physical barrier: Claudin-1, ZO-1, occludin ↑, Chemical barrier: colon crypt goblet cells ↑, Immune barrier: regulate Th17	*Bacteroidetes*, *Verrucomicrobia*, *Actinobacteria*, *norank_f_Muribaculaceae*, *Lactobacillus*, *Akkermansia* ↑ *Proteobacteria, Fusobacteria, Bacteroides, Escherichia-Shigella, Romboutsia, Fusobacterium* ↓	–	(Yan et al., 2022)
Kombucha	T2D	Physical barrier: ZO-1, claudin-1, occludin ↑ Chemical barrier: mucin 2 ↑,	*Lactobacillus*, *Butyricicoccus*, *Lachnospiraceae_NK4A136_group ↑* *Desulfovibrio*, *Escherichia-Shigella*, *Bacteroidetes* ↓	SCFA: acetate, butyrate ↑	(Xu et al., 2022)
Huazhi Rougan Formula	NAFLD	Physical barrier: ZO-1, occludin, claudin-2 ↑	*Lactobacillaceae*, *Bifidobacteriaceae*, *Clostruduaceae*, *Chostridiales, VadinBB60*, *Corynebacteriaceae*, *Solanales*, *Propionibacteriaceae*, *Micrococcaceae*, *Satphylococcaceae ↑*	BA: DCA, *β*-DCA, GDCA, LCA, isoLCA, 6-ketoLCA ↓	(Li et al., 2022a)
Linggui Zhugan Decoctions	Obesity IR	Physical barrier: occludin ↑	*Akkermansia*, *Ruminococcus torques*, *Erypelatoclostridium*, *Faecalitalea*, *Phascolarctobacterium ↑* *Collinsella* ↓	BA: regulate the expression of CYP7A1, CYP8B1, TGR5 and MRP2/3	(Ning et al., 2022)
Rhubarb extract	Constipation	Chemical barrier: goblet cells, mucin 2, TFF3 *↑*	Prevotellaceae_UCG-001 *Bacteroides ↑* Ruminiclostridium *Alistipes*, *Prevotellaceae_NK3B31_group, Ruminococcaceae_UCG-014 ↓*	SCFA: isovaleric acid, isobutyric acid ↑	(Gao et al., 2021)
*Ganoderma lucidum* Ethanol Extraction	Colitis	Physical barrier: ZO-1, occludin, claudin-1, claudin-3 *↑* Chemical barrier: mucin 2 *↑*	*Turicibacter, Bifidobacterium, Parabacteroides ↑* *Escherichia*_*Shigella, Bacteroides*, *Staphylococcus ↓*	–	(Li et al., 2022)
Evodiamine	UC	Physical barrier: occludin, claudin-1 *↑* Chemical barrier: mucin 2, TFF3 *↑* Immune barrier: Reg3-*γ*, Reg3-*β ↑*	*Firmicutes*, *L.acidophilus ↑* *Bacteroidetes*, *Verrucomicrobia* ↓	SCFA: acetate ↑ Trp ↑	(Wang et al., 2020a)
Ganoderma lucidum	Metabolic syndrome	Physical barrier: ZO-1 *↑* Chemical barrier: mucin 1, mucin 5 *↑*	*Clostridium* XIVa, XVIII, and IV, *Parabacteroides goldsteinii ↑* *Escherichia fergusonii*, *Prevotella, Fusobacterium, Selenomonas, Alistipes, Oscillibacter ↓*	SCFA: butyrate-activated PPAR-*γ* signaling ↑	(Wang et al., 2018)
Non-Alcoholic Components in Huangjiu	Alcohol liver disease	Physical barrier: ZO-1, occludin ↑ Immune barrier: Reg3-*β*, Reg3-*γ* ↑	*Lactobacillus*, *Faecalibaculum*, *Akkermansia* ↑	SCFA: acetate, butyrate ↑	(Yang et al., 2022b)
Gegen Qinlian Decoction	UC	Physical barrier: ZO-1, occuludin, claudin-1 ↑	Phylum level: *Firmicutes*, *Bacteroidetes ↑*, *Proteobacteria ↓* Class level: *Bacteroidia*, *Clostridia ↑*, *Gammaproteobacterial ↓* Order level: *Bacteroidales*, *Clostridiales ↑*, *Enterobacteriales ↓*	Trp: IPA (Indole-3-propionic acid), IAA (Indoleacetate), ILA (Indole-3-lactic acid), IAAld (Indole-3-acetaldehyde) ↑	(Wang et al., 2023)
*Antrodia cinnamomea*	Obesity	Physical barrier: ZO-1, occludin ↑ Immune barrier: Reg3g, lysozyme C ↑	*Streptococcus* spp., *Eubacterium* spp., *Eggerthella lenta*, *Clostridium* IV (*Clostridium methylpentosum*) *A. muciniphila ↑* Family level: *Ruminococcaceae*, *Lachnospiraceae ↓* Species level: *Clostridium scindens*, *Clostridium cocleatum* ↓	–	(Chang et al., 2018)
Sishen Wan	UC	Immune barrier: restore the balance of Th17/Treg	*Firmicutes* (phylum), *Lactobacillus ↑* *Bacteroidota* (phylum) ↓	SCFA: butyrate *↑*	(Wang et al., 2022d)
Bawei Xileisan	UC	Physical barrier: occuludin ↑ Immune barrier: restore the balance of Th17/Treg, IL-17A, IL-17, IL-22 *↓*	*Bacteroides*, *Lactobacillus ↑*	–	(Wen et al., 2016)
Shengjiang Xiexin Decoction	Antibiotic-Associated Diarrhea	Physical barrier: ZO-1 ↑ Chemical barrier: mucin 2, goblet cells ↑	*Bacteroides* spp*. Lactobacillus* spp. ↑ *Escherichia_Shigella* spp. *↓*	BAs: Ursodeoxycholic acid, deoxycholic acid, lithocholic acid ↑, Taurocholic acid *↓*	(Zhang et al., 2023)
*Hirsutella sinensis* mycelial polysaccharides	Obesity	Physical barrier: ZO-1 ↑ Immune barrier: T cells, IL-10 ↑, IL-1*β*, TNF-*α*, M1 ↓	*Parabacteroides_goldsteinii*, *Intestinimonas_butyriciproducens*, *Clostridium_cocleatum* ↑ *Shewanella_algae* ↓	–	(Wu et al., 2019b)
Gardenia jasminoides Ellis polysaccharide	Cholestatic liver injury	Physical barrier: villus structure of ileac epithelium ↑, ZO-1, occludin ↑	*Bacteroides ↑* *Enterobacteriaceae*, *Enterococcaceae ↓*	SCFA: acetate, propionate, butyrate ↑ BA: regulate the secretion of BAs, FXR, PXR ↑, the hepatic expression of metabolic enzyme Cyp3a11 ↑	(Fang et al., 2022)
Inulin and Lycium barbarum polysaccharides	T2D	Physical barrier: ZO-1, occludin ↑ Immune barrier: *γδ* IELs, TLR2^+^ <^i>^γδ </i>IELs *↑*	Phylum level: *Bacteroidetes*, *Cyanobacteria ↑* *Firmicutes*, *Dferribacteres*, *Tenericutes ↓* Genus level: *Bifidobacterium*, *Lactobacillus*, *Alistipes ↑* *Blautia*, *Desulfovibrio ↓*	SCFA: acetic acid, propionic acid, butyric acid, valeric acid ↑	(Lu et al., 2021)
turmeric polysaccharides	DSS-induced UC	Physical barrier: ZO-1, occludin ↑	*Clostridia-UCG-014*, *Lactobacillus*, *Akkermansia*, *Bacteroides* ↑ *Firmicutes ↓*	SCFA: acetate, propionate, butyrate, valeric acid ↑ Trp: IAA indole-3-acetaldehyde (IAAId)↓, indole-3-acetamide (I3AM) ↓	(Yang et al., 2021a)
*Rosa roxburghii* Tratt fruit polysaccharide	Obesity-induced Colitis	Physical barrier: ZO-1, caludin-1, occludin *↑*	*Bacteroidetes, Muribaculaceae, Lachnospiraceae, Oscillospiraceae, Akkermansiaceae, Ruminococcaceae, Eggerthellaceae, Bacteroidaceae, norank_o Clostridia_UCG-014, Tannerellaceae, Saccharimonadaceae, Prevotellaceae ↑* *Firmicutes*, *Actinobacteriota*, *Verrucomicrobiota*, *Erysipelotrichaceae*, *Lactobacillaceae*, *Atopobiaceae*, *Desulfovibrionaceae ↓*	SCFA: acetate, propionate, butyrate *↑*	(Wang et al., 2022c)
*Polygonatum kingianum* Polysaccharide	T2D	Physical barrier: ZO-1, occludin *↑*	*Bifidobacterium*, *Streptococcus*, *Ruminococcus*, *Anaerovibrio, Roseburia* ↑ *Proteobacteria*, *Lactobacillus, Psychrobacter* ↓ *Bacteroidetes* **/** *Firmicutes ratio ↑*	SCFA: acetate, propionate, butyrate ↑	(Yan et al., 2017)
*Poria cocos* Polysaccharide	NAFLD Antibiotic-Associated Diarrhea DSS-induced UC	Physical barrier:ZO-1, occludin *↑* Immune barrier: M1, IL-6, IL-1*β ↓*, M2 *↑*	*Bacillota, Ruminococcus_gnavus Parabacteroides_distasonis, Akkermansia_muciniphila*, *Clostridium_saccharolyticum, Lactobacillus_salivarius, Salmonella_enterica, Mucispirillum_schaedleri ↑* *Verrucomicrobiota Pseudomonadota ↓*	SCFA: GPR41, GPR43 *↑*	(Ye et al., 2022; Xu et al., 2023)

In “Improved barrier and corresponding altered indicators” and “Variation of gut microbial metabolites” parts, ↑ means the expressions of indicators or metabolites took on an increased trend, and ↓ means the expressions of those took on a decreased trend.

In “Variation of gut microbiota” part, ↑ means the abundance of the mentioned gut microbiota took on an increased trend, and ↓ means the abundance of those took on a decreased trend.

TCM indirectly affects the gut microbiota mainly in two ways. Firstly, it influences the growth environment of the bacteria, impacting their composition. Secondly, TCM can modify the transit time in the gastrointestinal tract. Gastrointestinal pH plays a critical role in modulating the bacteria density and its metabolite such as SCFAs. The population of the butyrate-producing *Roseburia group* presents a declining trend when pH is altered from 5.5 to 6.5, and paralleled concentration of butyrate presents a similar tide (Walker et al., 2005). Besides, the biological activity of enzymes can be impacted as well. TCM can also shape the bacteria-living environment from another aspect by modulating the immune responses in the gastrointestinal tract. For example, Fei-Xi-Tiao-Zhi-Fang can increase the sIgA in the model rats’ intestine, which may be the result of modulation of different microbial genera, as the abundance of *g_Lactobacillus* was observed to negatively change compared to sIgA (Liu et al., 2018b). Increased slgA provided a barrier against noxious substances, potentially guaranteeing other beneficial microbiota density and activity. In addition to the indirect reactions mentioned above, TCM can also enhance gut barrier function and increase nutrients for beneficial microbiota (Kamada et al., 2013; Lu et al., 2018). Besides, gastrointestinal transit time can affect gut microbiota metabolism. Transit time determines how long TCM compounds interact with gut microbiota, influencing their composition. Shorter transit time reduces metabolite production. A study has shown that TCM decoctions can modify transit time by influencing the interstitial cells of Cajal (Hwang et al., 2015).

### 3.3 TCM regulate gut microbial metabolites

When orally administered into the gastrointestinal tract, TCM can affect the metabolism of the gut microbiota and change the type and content of its metabolites (SCFA, BA, Trp, etc.). The reactions producing gut microbiota metabolites are as follows: (1) TCM can be the direct source of gut microbiota metabolite by interacting with gut microbiota, (2) TCM regulates the gut microbiota composition and therefore influence the production of metabolite, (3) TCM alters the activity and abundance of the enzymes essential for gut metabolite production, (4) an aggregation of these effects.

#### 3.3.1 SCFAs

Known as the most exhaustively studied microbiota metabolite, SCFAs are a group of fatty acids that typically contain no more than six carbon atoms. SCFAs include formate, acetate, propionate, butyrate, valerate, and their corresponding isomeric forms. SCFAs mainly originate from the fermentation of non-digestible carbohydrates through a sequence of enzymatic reactions mediated by gut microbiota in the colon. The majority of SCFAs undergo enzyme-associated decomposition reactions to be metabolized by certain gut microorganisms such as the phylum *Bacteroidetes* and *Firmicutes*, which can encode genes to degrade polysaccharides (Xu et al., 2013). As the most comprehensively studied SCFA, butyrate can be obtained after the reaction that butyryl-CoA is converted into butyrate by phosphotransbutyrylase and butyrate kinase. What is worth noticing is that by the butyryl-CoA: acetate CoA-transferase route, gut microbiota can also convert butyryl-CoA into butyrate (Duncan et al., 2002; Louis et al., 2004). In terms of the production of other SCFAs, acetate arises from pyruvate via acetyl-CoA as well as Wood-Ljungdahl pathway, and propionate can derive from the reduction of lactate in acylate pathway. And an array of gut microbiota partakes in the reactions mentioned above (Scott et al., 2006; Rey et al., 2010; Louis et al., 2004).

Via acting as signaling molecules, SCFAs are equipped with quantities of functions such as modulation of gut integrity, gut hormone production, oxidative stress, appetite, and immune function (Feng et al., 2022). SCFAs can also serve as staple and preferred energy resources for the gut microbiota and host. SCFAs can either act as agonists for G protein-coupled receptors (GPRs) or exert suppressive influence on nuclear 1 histone deacetylase (HDAC) (HDAC1 and HDAC3) to affect the homeostasis. Through interfaces with GPR41 (free fatty acid receptor 3 or FFAR3) and GPR43 (free fatty acid receptor 2 or FFAR2) (Stoddart et al., 2008) as ligands, SCFAs are eligible of promoting the secretion of insulin by regulating the release of intestinal peptide hormones glucagon-like peptide-1 (GLP-1) and peptide YY (PYY) in pancreatic *β* cells, and hence can bring a restoration to aberrant insulin sensitivity and gluconeogenesis (Christiansen et al., 2018; Larraufie et al., 2018; Priyadarshini et al., 2018). When activating nod-like receptor family pyrin domain containing 3 (NLRP3), SCFAs can decline the amount of pro-inflammatory makers while they can enhance the proliferation and function of pancreatic *β* cells by suppressing the activity of HDAC (Zhang et al., 2021; Zhang et al., 2022b). Moreover, a positive influence on immunology responses can also be produced through inhibition of HDAC by the SCFAs (Koh et al., 2016; Zhang et al., 2021). A few SCFAs, most of which are acetate and propionate, can directly affect the adipose tissue, brain, and liver, contributing to a sequence of beneficial effects on the host (Koh et al., 2016).

Both the increase and decrease of SCFAs abundance can be triggered by TCM. Polysaccharides can directly act as the abundant source of SCFAs. These macromolecular substances escaping from digestion and absorption will be turned into SCFAs by the fermentation of gut microbiota (Zhang et al., 2022b). TCM can regulate the levels of SCFAs by affecting gut microbiota and enzymes. For example, Mulberry leaf has been reported to reverse the decrease of SCFAs induced by cyclophosphamide, and it can establish a cozy intestinal microenvironment for butyrate-producing bacteria which has been damaged by inflammatory cytokines before (Chen et al., 2021a). Additionally, the abundance of SCFAs exhibited a declining trend with the utilization of Pueraria lobata in antimicrobiota-associated diarrhea mice, and this treatment also altered the richness of the gut microbiota associated with SCFAs production (Chen et al., 2020). Furthermore, Shenling Baizhu powder can increase the abundance of *Bifidobacterium* and *Anaerostipes*, which can produce promotive metabolic impact on metabolism of SCFAs (Zhang et al., 2018b). Apart from modulating the levels of gut microbiota, TCM can also alter the metabolism of SCFAs by regulating the expression and activity of SCFA biosynthesis-associated enzymes. It has been demonstrated that berberine can significantly augment the formation of acetate, propionate and butyrate in rats, where prominent enzymes including Butyryl-CoA:acetate-CoA transferase (BUT), acetate kinase, and methylmalonyl-CoA decarboxylase were observed to increase (Wang et al., 2017a). And when the expressions of BUT and crotonyl-CoA and butyryl-CoA (viewed as butyrate synthesis-associated key substances) were promoted by berberine, blood lipid and glucose levels were consequently decreased (Wang et al., 2017b).

#### 3.3.2 BAs

Deriving from cholesterol in the hepatocytes, BAs are a series of metabolites with a steroid structure and can be classified into two categories according to their resource (Fiorucci and Distrutti, 2015): primary BAs and secondary BAs. BAs play important roles in attenuating hyperlipidemia (Kong et al., 2021) and ameliorating systematic inflammation (Wei et al., 2018). Moreover, BAs are involved in correcting abnormal insulin sensitivity and glucose metabolism, while also serving as signaling molecules that facilitate inter-tissue communication between the liver and other organs (Perino et al., 2021). Cholesterol can be converted into primary BAs in two ways: the classic pathway (responsible for most production of primary BA) and the alternative pathway, which contains sophisticated successive reactions (Cheng et al., 2022a). Primary BAs can be transformed into bile salts by bile acid cholyl-CoA synthetase and bile acid-CoA: amino acid Nacyltransterase before entering intestine from gallbladder (Martinot et al., 2017). Bile salts will undergo deconjugation by bile salts hydrolase and then, free BAs can be released, which is the premise of secondary BAs formation. And many gut microbiota such as *Lactobacillus*, *Bifidobacteria*, and *Bacteroides fragilis* have been proved to produce bile salts hydrolases (Cheng et al., 2022a). The majority of free BAs can be reabsorbed and then transported back to liver, which is known as enterohepatic circulation. The rest of free BAs can be transformed into secondary BAs. The formation of secondary BAs involves a sequence of enzymatic reactions such as deconjugation, dehydroxylation and reconjugation, which are mediated by gut microbiota (Ridlon et al., 2014). In addition, gut microbial enzymes such as 7*α*-dehydroxylase and bacterial stereospecific hydroxysteroid dehydrogenases have been recognized as important factors for the formation of secondary BAs (Cheng et al., 2022a).

BAs can regulate lipids, amino acids, glucose metabolism by activating quantities of BA receptors including the cell surface receptor such as Takeda G-protein receptor (TGR5) and the intracellular receptors such as vitamins D3 receptor (VDR), farnesoid X receptor (FXR), pregnane X receptor and constitutive androstane receptor (Fiorucci and Distrutti, 2015; Xie et al., 2020). Disturbance of BAs receptors activity will trigger mass of host diseases including obesity, T2D, NAFLD, nonalcoholic steatohepatitis (NASH) and colon cancer (Jia et al., 2021).

TCM compounds can regulate BA metabolism via affecting enzymes and receptors involved in BA metabolism. These modulations are partly linked to the control of gut microbiota. For instance, the administration of *Sophora alopecuroides* has been shown to ameliorate colitis in DSS-induced mice, with concurrent changes in gut microbiota composition. Furthermore, this ameliorative effect on colitis was associated with an increase in BA levels (Jia et al., 2020). In another case of NAFLD mice, the extract of *Penthorum chinense* Pursh. was found to reduce the abundance of bacteria producing bile acid salt hydrolase (*Clostridium_IV*, *Clostridium_XIVb*, and *Lactobacillus*), resulting in decreased BA hydrolysis. This led to the accumulation of tauro-conjugated bile acids, which then decreased the levels of intestinal FXR and FGF15. Consequently, BA synthase activity was activated, promoting the conversion of cholesterol to bile acids and ultimately reducing cholesterol levels (Li et al., 2022d). Besides, in a study examining the therapeutic effects of Cryptotanshinone on mice with radiation-induced lung fibrosis, the levels of specific BAs, such as DCA and LCA, which activate FXR, were positively correlated with the relative abundance of *Lachnospiraceae* and *Akkermansia*. The decrease in *Lachnospiraceae* bacteria may reduce BA metabolism in cancer cachexia, while *Akkermansia* can enhance the production of coenzyme A and increase the synthesis of conjugated primary BAs. Hence, Cryptotanshinone may regulate BA metabolism by increasing the abundance of *Lachnospiraceae* and *Akkermansia* (Li et al., 2023). Other than that, Pu-er tea can increase BAs synthesis-associated enzymes in high-fat diet mice, including oxysterol 7*α-*hydroxylase (CYP7B1), mitochondrial sterol 27-hydroxylase (CYP27A1), and cholesterol 7*α*-hydroxylase (CYP7A1) (Huang et al., 2019).

#### 3.3.3 Trp

Trp is an amino acid whose metabolism in the gut mainly covers three types: Trp can either be transformed into kynurenine, kynurenic tryptophan 2,3-dioxygenase and indoleamine 2,3-dioxigenase (kynurenine pathway) or be converted into serotonin and melatonin by Trp hydroxylase 1 (serotonin pathway) (Agus et al., 2018). Besides, Trp can be directly transformed into several substances by gut microbiota, such as indole and indole acid derivatives (indole propionic acid, indole acetic acid) (Agus et al., 2018). These metabolites can act as ligands for Aryl hydrocarbon receptor (AhR), and AhR signaling is crucial for regulating gut immune response and intestinal health (Agus et al., 2018). The metabolism of Trp has been proved to be of great value for the health of the host. As an example, the Trp-derived metabolites indolepropionic acid and indole were found to decrease in the serum of mice with DSS-induced colitis (Alexeev et al., 2018). And other diseases, including NAFLD, Crohn’s disease, obesity, stroke, mucosal candidiasis, chronic kidney disease, autism spectrum disorder, and Alzheimer’s disease, are also proved to be associated with impaired metabolism of Trp (Liu et al., 2022).

Studies demonstrating the potential of TCM to influence the metabolism of Trp abound. Remarkably increased Trp and lysine have been discovered in diabetic rats after the treatment of tea polysaccharide (Li et al., 2020a). Turmeric polysaccharides can recover damaged gut microbiota composition and increase indole-3-acetaldehyde (IALD) and indole-3-acetic acid levels (Yang et al., 2021c). Ganoderma lucidum polysaccharide can decrease the amount of indole lactate in colon while increasing the levels of *Roseburia*, *Oxalobacter*, *Akkermansia*, and decreasing those of *Coprococcus*, *Eubacterium*, *Mogibacterium*, *Coprobacillus*, and *Ruminococcus* (Jin et al., 2019). Considering gut bacteria such as *Lactobacillus* and *Akkermansia* are capable of promoting Trp metabolism (Gao et al., 2021; Yin et al., 2021), it is quite possible that the therapeutical effects of Ganoderma lucidum polysaccharide was achieved by modulation of microbial Trp metabolism.

## 4 TCM enhance gut barrier via regulating gut microbiota

TCM usually exerts a comprehensive effect on multiple gut barriers instead of on a sole one. On one hand, TCM can regulate gut microbiota and its metabolites to enhance the gut barriers. On the other hand, gut barrier can be directly regulated by TCM.

### 4.1 Physical barrier

The damaged physical barrier is associated with a number of diseases such as inflammatory bowel disease, leaky gut, celiac disease, graft-versus-host-disease, diabetes, multiple sclerosis, and autism (Turner, 2009). Extraneous and endogenous opportunistic pathogens can damage the gut physical barrier, which in turn leads to increased permeability of the gut. LPS has been proved to be a detrimental factor to intestinal homeostasis, for it can give rise to enterocyte autophagy and destruction of gut barrier (Nguyen et al., 2013; Feng et al., 2018). LPS are structural components of the cell wall in gram-negative bacteria, and are derived from the death and lysis of these bacteria (Zhang et al., 2022a). The increased permeability of physical barrier will cause high LPS translocation in the blood stream, a condition known as metabolic endotoxemia (Cani et al., 2007). SCFAs can defend the barrier against the intestinal damage induced by LPS (Jiang et al., 2017). SCFAs can also consolidate the assembly of TJ proteins via activating AMP-activated protein kinase and promoting transcription of claudin-1 and expression of ZO-1 (Feng et al., 2018).

**Figure 3:**
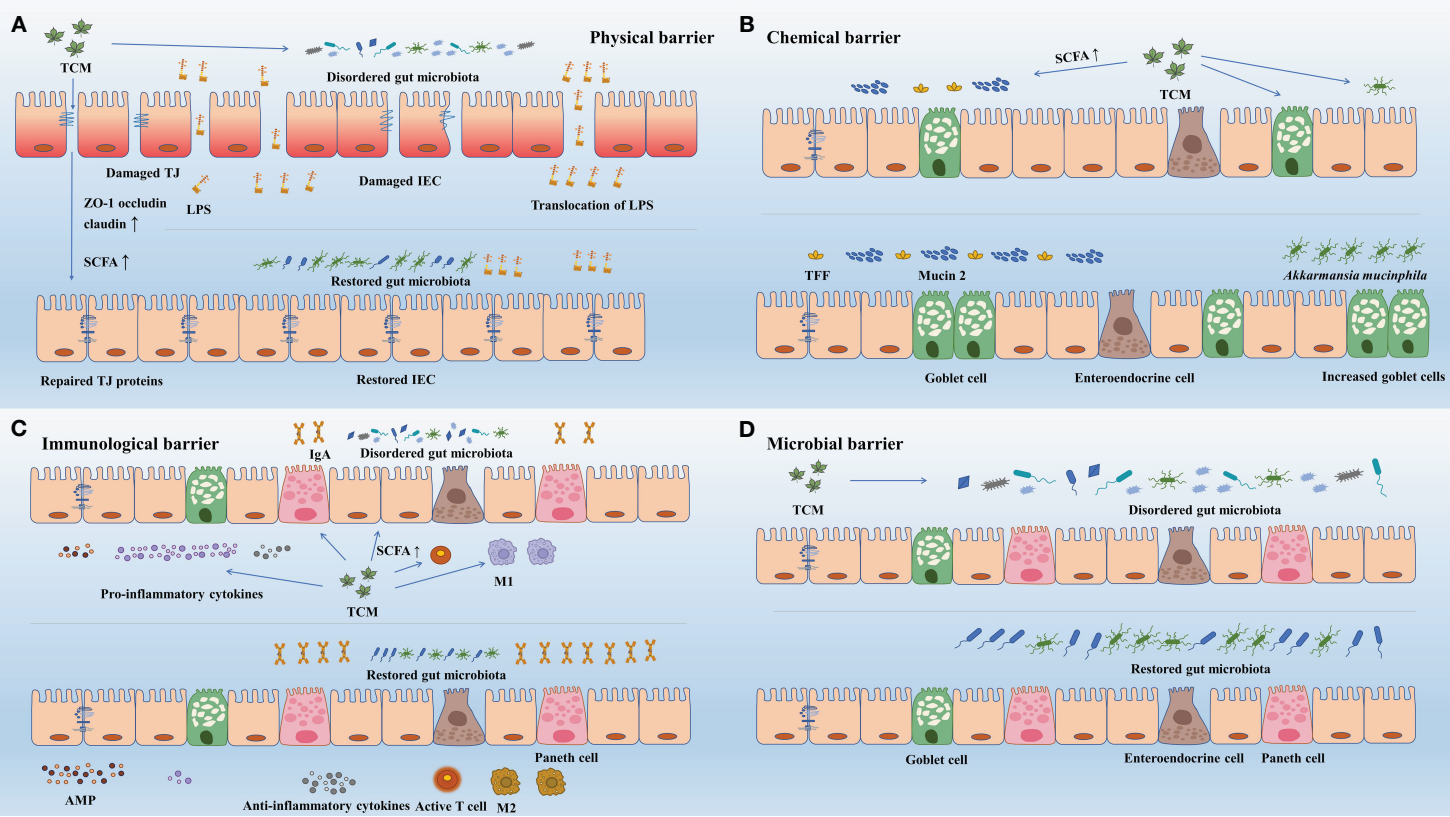
TCM enhance gut barriers via regulating gut microbiota and its metabolites. **(A)** Via regulating gut microbiota and its metabolites, TCM improved TJ structure (ZO-1, occludin, claudin) and restored damaged intestinal epithelial cells, and thus improved gut physical barrier. The enhanced physical barrier further prevents translocation of pathogens. **(B)** Via regulating gut microbiota and its metabolites, TCM improved the abundance of goblet cells and increased the expression mucin 2 and TFF, and thus strengthened the gut chemical barrier. **(C)** Via regulating gut microbiota and its metabolites, TCM acted on immune cells to increase the expression of antimicrobial peptides (e.g., sIgA), enhanced the synthesis and release of anti-inflammatory cytokines and inhibited that of pro-inflammatory cytokines. Besides, TCM helped to activate immune cells (e.g., T-cells) and facilitate the transformation of immune cells (e.g., M1 to M2). The aggregation of these effects enhanced the gut immunological barrier. **(D)** TCM directly regulated the community structure, the diversity and richness, and the specific abundance of gut microbiota to restore damaged gut microbial barrier. AMP, antimicrobial peptide; IEC, intestinal epithelial cell; IgA, immunoglobulin A; LPS, lipopolysaccharide; M1, macrophage 1; M2, macrophage 2; SCFA, short chain fatty acid; TCM, Traditional Chinese Medicine; TFF, trefoil factor; TJ, tight junction; ZO-1, zonula occludens 1.

TCM can consolidate the physical barrier via modulating gut microbiota composition and gut microbiota metabolites such as SCFAs (**Figure 3**). *Polygonatum kingianum* polysaccharide has been documented to attenuate glucose and lipid metabolic disorder by repairing the gut physical barrier in high-fat diet rats. After its administration, the levels of ZO-1 and occludin were increased and the translocation of LPS was suppressed. And the increased levels of acetate and propionate and regulated *Lactobacillus*, *Psychrobacter*, *Bacteroides, Allobaculum, Blautia*, and *Phascolarctobacterium* also helped to fortify the gut physical barrier (Yan et al., 2017). Besides, Shugan Xiaozhi Decoction, a formula that can attenuate NAFLD in high-fat diet-induced rats, can also inhibit the translocation of LPS to avoid inflammation damage and increase ZO-1, occludin to fortify the gut barrier. This effect may be correlated with the lifting levels of *f_Prevotellaceae* and declining those of *f_Desulfovibrionaceae* and *f_Erysi-Pelotrichaceae* (Yang et al., 2022a). Mulberry leaf polysaccharide (MLP) can enhance the gene expression of occludin 1, ZO-1 and claudin 5 and relive intestinal villus damages. And the upregulation of the richness of butyrate-producing bacteria and downregulation of the *Eubacterium*-to-*Bacteroidetes* ratio can also be achieved by MLP to activate intestinal immunity and strengthen physical barrier. Ultimately, all these restorations boosted the recovery from immunosuppressed symptoms in cyclophosphamide-treated mice (Chen et al., 2021a). Another example is Astragluse polysaccharide, which can modulate the levels of *Anaerofustis, Anaeroplasma, Anaerotruncus, Lachnospira*, and *Ruminococcus*, then may potentially affecting the metabolism of butyrate (Li et al., 2022c). Apart from that, Astragluse polysaccharide was also found to fortify the gut physical barrier by increasing villus height and crypt depth to strengthen mucosal immunity in broiler chickens (Yang et al., 2021b). Thus, it is highly possible that the reinforcement of gut barrier was induced by increased SCFAs. But there exist exceptions. For example, in DSS-induced mice, the declining trend in mRNA expression of ZO-1, claudin-1 and occludin was reversed by Puerarin, while the amount of SCFAs showed a contradictory variation (Jeon et al., 2020). Hence, when the host gut physical barrier is enhanced, the variation of the TJ proteins expression and the SCFAs levels are not always consistent. But mounts of facts remind us that there might be a specifically causal linking between the enhancement of TJ proteins and SCFAs. To figure out the detailed mechanism, more studies need to be carried out.

### 4.2 Chemical barrier

Mucin 2 plays an important role in maintaining the function of the gut chemical barrier. Bacteria are found to penetrate down into the crypts and epithelium in mice without mucin 2, and the bacterial invasion has been proved to be capable of triggering inflammation, diarrhea, rectal bleeding, colitis, and risen risk of colon cancer (Van der Sluis et al., 2006; Petersson et al., 2011). SCFAs can lift the levels of mucin 2. Butyrate, one of the most common SCFAs, is closely connected to the protection of intestinal epithelial cells and metabolism in the gut. More vitally, butyrate can enhance the mucin 2 gene expression in a dose-associated way, and this effect is achieved by mediation via AP-1 and acetylation/methylation of histones at the mucin 2 promoter (Burger-van Paassen et al., 2009). Apart from butyrate, acetate, and propionate can also positively alter the levels of mucin 2 (Burger-van Paassen et al., 2009).

Defects of chemical barrier in UC patients are commonly observed. These defects are attributed to two factors: the active inflammatory environment, which reduces the number of goblet cells, and long-term changes in mucin secretion that persist even when inflammation is absent (Singh et al., 2022). TCM has the potential to restore damaged chemical barrier by improving gut microbial metabolites. A specific example is the administration of puerarin in mice with UC induced by DSS, which has been shown to rebuild the damaged barrier and attenuate lesions. Puerarin ingestion improved the production of SCFAs and prevented the overgrowth of mucin-utilizing microbiota. It also enhanced goblet cell differentiation, secretion, and restored the thickness of the mucus layer. These effects likely contributed to the restoration of mucus and goblet cell function, and the changes in SCFAs and bacteria community may be the underlying causes (Wu et al., 2020).

In addition to mucin 2, trefoil factor 3 (TFF3) is another component of the mucus layer secreted by goblet cells that aids in mucosal repair and content renewal. TFF3 promotes viscosity by binding to the von Willebrand factor C domain of mucin 2, thereby enhancing mucus integrity (Tomasetto et al., 2000). Cinnamaldehyde has been proved to directly promote the expression of TFF3 and mucin 2 in early-weaned rats (Qi et al., 2022). The richness of *Akkermansia*, *Bacteroides*, *Clostridium III*, *Psychrobacter*, and *Intestinimonas* were increased and those of *Ruminococcus* and *Escherichia/Shigella* were declined in the experiment. As a result, the repaired gut chemical barrier effectively inhibited inflammatory response in the intestinal tract. Furthermore, it has been documented that Rhubard extract (RE) was conducive to promoting goblet cells to increase the density of mucin 2 and TFF3, then strengthening the concentration, viscosity, and function of the mucus layer in constipation mice model induced by diphenoxylate (Gao et al., 2021). RE also increased the level of *Bacteroides*, decreased that of *Alistipes*, and lifted the concentration of butyric acid, isobutyric acid, and isovaleric acid. Apart from that, it is important to note that there is a potential relationship between mucin 2 and TFF3. Supplementation of mucin 2 has been found to restore decreased mRNA levels of TFF3 in rats with acute necrotizing pancreatitis (Huang et al., 2023). However, the regulation in gut microbiota and its metabolites by TCM may be the key reason for the improvement in mucus. Further research is needed to fully understand the interplay between gut microbiota, its metabolites, mucin 2, TFF3, and their effects on mucus.

As one of the bacteria that benefit from TCM, *Akkermansia* has gained attention for its well-established role as a significant contributor to mucus maintenance and its anti-inflammatory properties (Shin et al., 2019; Leong et al., 2022). It shows an excellent ability to multiply the abundance of intestinal goblet cells and increase the levels of mucin 2, and can upregulate the mucin gene and the regeneration of mucin, which eventually contribute to normalization and incrassation of the mucus layer (Tropini et al., 2018; Shin et al., 2019). Qiwei Baizhu Decoction, a classic herbal formula, has been widely used in clinical practice to treat diarrhea by repairing the mucus barrier. It has been observed to enhance the expression of mucin 2 in juvenile rats with diarrhea. This positive effect may mainly be attributed to the increased abundance of *Akkermansia* and the simultaneous increase in the expression of SCFAs stimulated by the decoction (Sun et al., 2021b). While *Akkermansia* utilizes mucin as an energy source, it is more accurate to describe it as a mucin utilizer rather than a mucin degrader. Unlike pathogens or other mucin-degrading bacteria, *Akkermansia* utilizes mucins by specifically degrading O-glycans without disrupting the integrity of the inner mucus layer barrier. This allows for a balanced rate of mucus utilization in coordination with mucus synthesis, resulting in a dynamic and stable mucus structure (Suriano et al., 2022). In contrast, pathogens have the ability to break down the polymeric network of mucins, leading to the collapse of the mucus structure (Luis and Hansson, 2023). However, there are still other underlying mechanisms that remain unknown and require further investigation.

### 4.3 Immunological barrier

SCFAs can partake in immune responses, change the expression of AMP, and consolidate the gut immune barrier in multiple ways. SCFAs can directly interact with GPR43 to upregulate the production of Reg3-*γ* and *β*-defensins in IECs, which relies on the activation of the mammalian target of rapamycin via GPR43 (Zhao et al., 2018). Besides, it has also been studied that SCFAs can activate the NLRP3 inflammasome through G-protein coupled receptors signaling, enhancing the defense against intestinal inflammation (Macia et al., 2015). SCFAs can regulate the differentiation of Treg (Arpaia et al., 2013). Additionally, SCFAs can suppress macrophage 1 (M1) and facilitate macrophage 2 (M2) polarization to enhance intestinal and host immune functions (Wang et al., 2020b). M1 is conducive to the recruitment of pro-inflammatory factors such as IL-1*β*, IL-6, and TNF-*α*, whereas M2 shows anti-inflammatory and tissue-repairing function (Biswas et al., 2006; Gabrilovich et al., 2012). Apart from gut microbial metabolites (SCFAs), gut microbiota is also indispensable for maintaining the function of the gut immune barrier. It has been found that there exist few AMPs (such as C-type lectin Reg3-*γ* and Reg3-*β*) in Germ-free mice compared with normally raised ones (Hooper et al., 2003; Cash et al., 2006; Vaishnava et al., 2011). Certain gut microbiota such as *Clostridium* spp., *Schaedler*, and *Bacteroides fragilis* have been proved to shift immune responses through promoting the development of certain subtypes of lymphocytes (Takiishi et al., 2017).

TCM can modulate the gut microbiota composition and its metabolites to complete the repairment and enhancement of the gut immune barrier (**Figure 3**). For example, the relative abundance of *Bifidobacterium*, a bacterium responsible for SCFAs production, showed an increasing trend with the administration of BuFei-JianPi-fang (BJF) in chronic obstructive pulmonary disease model rats (Mao et al., 2022). And the BJF also reinforced the gut immune barrier via counteracting the declined expression of sIgA and SCFAs in the colon mucus layer and downregulating NLRP3, caspase-1, IL-1*β*, and IL-18 levels, thereby delaying the aggravation of inflammation in the host (Mao et al., 2022).

The interplays between TCM and gut microbiota can also modulate the Paneth cell and intestinal macrophages in the intestine. Poria cocos polysaccharide (PCP) showed the ability to increase the abundance of Paneth cell and SCFA-producing bacteria*: Bacteroides intestinihominis*, *Butyricicoccus pullicaecorum*, *Lactobacillus johnsoni*, *Bifidobactrium choerinum*, and *Eubacterium* spp. in Apc^Min/+^ mice (Yin et al., 2022). PCP also suppressed M1 macrophages and facilitated M2 macrophages to increase the anti-inflammatory factors such as IL-4, IL-10, and IL-13 to enhance the fight against the intestinal side effects of 5-fluorouracil treatment (Yin et al., 2022). Another example is traditional Patchouli Essential oil (PEO) and its derivatives, they can increase the levels of phylum *Firmicutes* and *Akkermansia muciniphila* and decrease those of phylum *Bacteroidetes* in Apc^Min/+^ mice (Leong et al., 2022). Apart from that, the expression of SCFA receptors (GPR41, GPR43, and GPR109a) and Paneth cells were augmented and M1 macrophages were directly shifted to M2 macrophages by PEO and its derivatives (Leong et al., 2022). As a result of these joint regulations, the gut immune barrier was enhanced, resulting in the improvement of the immunity and metabolism of the host. Another example is polysaccharides from fermented coix (PFC), which can enrich the abundance of *Bacteroidetes* and decrease those of *Tenericutes*, *Firmicutes Anaeroplasma, Oscillospira, Peptococcaceae* and *Ruminococcus* in BALB/c mice exposed to high relative humidity and thus regulate the levels of SCFAs (acetic acid, propionic acid, butyric acid, isobutyric acid, valeric acid and isovaleric acid) (Wang et al., 2022a). As the gut microbiota was regulated by PFC, so were Th1/Th2 cytokines (Wang et al., 2022a), and these two parts collaborated on restoring gut immune function and circulating nitrogen disorder of the host. Altogether, to strengthen the gut immune barrier, TCM can modulate the secretion of AMP, proinflammatory and anti-inflammatory cells and cytokines. And the occurring of the reactions mentioned above usually coincides with the modulation of gut microbiota and its metabolites.

### 4.4 Microbial barrier

As the core of the gut microbial barrier, the composition of the gut microbiota community is essential to ensure the efficacy of TCM. It has been documented that deviated gut microbiota cannot offer the amelioration of steatohepatitis and resistance to colonization of extraneous pathogens. For example, Pien Tze Huang (PTH), a TCM with the function of alleviating NASH, failed to provide mice which are fed with antibiotics cocktail with protection against NASH (Zeng et al., 2024).

TCM usually directly modulates the richness of multiple gut microbiota to enhance gut barrier function (**Figure 3**). For example, a study has revealed that curcumin plays an important role in preventing and ameliorating gut microbial dysbiosis in diabetic rats, whose administration can decrease the abundance of *Enterobacterales* and *Firmicutes* and lift those of *Bacteroidetes* and *Bifidobacterium* (Huang et al., 2021). *Bifidobacterium* can form the biofilm on the surface of the intestinal mucosa, and regulate microenvironment and microecological balance of intestine, therefore contributing to the repairment of gut barrier (Schwiertz et al., 2010). Another example is that Sini Decoction can enhance the gut microbial barrier by lifting the abundance of *Bacillus coagulans*, *Lactobacillus*, *Akkermansia muciniphila* and *Bifidobacterium* in DSS-induced colorectal cancer mice (Wang et al., 2021). The similar result was also repeated by Ge Gen Qin Lian Decoction (GQD) in colorectal cancer patients, which promoted the abundance of *Bacteroides*, *Akkermansia* and *Prevotella* and decreased those of *Megamonas* and *Veillonella* (Li et al., 2020c). Via modulating gut microbiota composition, both of these two decoctions postponed the development of colorectal cancer (Li et al., 2020b; Wang et al., 2021).

Furthermore, the gut microbiota modulated by TCM can produce SCFAs, which lower the pH of the intestines, thereby inhibiting the growth of harmful microorganisms. Moreover, these TCM-modulated microbiota outcompete pathogens for oxygen and nutrients, preventing their overgrowth and invasion into underlying tissues, including the bloodstream (Hebbandi Nanjundappa et al., 2017). The function of lifting the abundance of SCFA-producing bacteria has been revealed in the Baicalin treatment in spontaneously hypertensive rats (Wu et al., 2019a). And the levels of acetic acid, propionic acid, butyric acid, isobutyric acid, valeric acid, and isovaleric acid were observed to increase. As a result, the gut microbial barrier was enhanced and the ulcerative intestinal lesions in rats were effectively relived.

TCM inclines to produce a comprehensive and protective function on multiple barriers instead of a sole one. GQD helped to promote other gut barrier function and inhibit the development of intestinal inflammation by altering gut microbiota composition in colorectal cancer patients. Because GQD can lift the expression of ZO-1 and occludin, inhibit the NF-*κ*B inflammatory signaling pathway, reduce the inflammatory factor TNF*-α* in blood and decrease tumor tissues (Li et al., 2020c). Furthermore, Inulin (INU) and Lycium barbarum polysaccharide (LBP) can lift the abundance of *Bifidobacterium* and *Lactobacillus* in T2D model rats (Lu et al., 2021). These two bacteria can promote CD8 T-cells activation and costimulation and strengthen DC defensive function (Ljungh and Wadström, 2006; Sivan et al., 2015). Besides, the enhanced gut microbial barrier and increased SCFAs (acetic acid, propionic acid, butyric acid, and valeric acid) induced by INU and LBP have been shown to be positively correlated with the promotion of ZO-1 and occludin, as well as the upregulation of *γδ* IELs and TLR2^+^ *γδ* IELs. These changes collectively improved both the gut physical and immune barrier, subsequently relieving systemic inflammation in the host. Hence, by the interfaces between TCM and microbiota, both gut microbial barrier and other gut barriers can be consolidated.

## 5 TCM alleviate diseases via regulating gut microbiota and gut barrier

An array of diseases, such as celiac disease, enteric infections, Crohn’s disease, UC (Turner, 2009), T2D (Ma et al., 2019) and NASH (Albillos et al., 2020), have been documented to be correlated with the dysbiosis of gut microbiota, and TCM compounds have presented excellent ability to maintain the intestinal homeostasis by regulating gut microbiota and further consolidating the gut barriers (Wu et al., 2018). In **Tables 1**, **2**, we have listed the effects of TCM on gut microbiota, its metabolites, gut barriers, and diseases.

**Table 2:** Therapeutic efficacy of TCM compounds on gut barrier, gut microbiota, and gut microbiota metabolites.

TCM	Disease	Improved barrier and corresponding altered indicators	Variation of gut microbiota	Variation of gut microbial metabolites	Reference
Lycium ruthenicum Anthocyanins	Obesity	Physical barrier: JAMA, ZO-1, occludin, claudin-1 ↑ Chemical barrier: goblet cells, mucin 2 ↑	*Ruminococcaceae*, *Muribaculaceae*, *Akkermansia*, *Ruminococcaceae_UCG-014*, *Bacteroides* ↑ *Helicobacter Desulfovibrionaceae* ↓	SCFA: acetate, propionate, isobutyrate, butyrate, isovalerate valerate↑	(Tian et al., 2021)
Curcumin	T2D	Physical barrier: occludin, ZO-1 ↑	*Bacteroidetes Bifidobacterium* spp. *↑* *Enterobacterales Firmicutes* ↓	–	(Huang et al., 2021)
*Ramulus Mori* (Sangzhi) Alkaloids	T2D Obesity NAFLD	Physical barrier: ZO-1 ↑	*Bacteroidaceae*, *Erysipelotrichaceae*, *Verrucomicrobia*, *Bacteroides*, *Faecalibaculum*, *Allobaculum* ↑ *Rikenellaceae*, *Desulfovibrionaceae*, *Aerococcaceae*, *Alistipes*, *Desulfovibrio*, *Aerococcus* ↓	SCFA: acetate, propionate ↑, butyric, isobutyric (propionic acid-2-methyl), pentanoic, isopentanoic (butanoic acid-3-methyl), hexanoic, isohexanoic (pentanoic acid-4-methyl) acids ↓	(Liu et al., 2021)
Andrographolide	T2D	Physical barrier: occludin, ZO-1 ↑ Chemical barrier: mucin 2 ↑	*Prevotella Adlercreutzia, A,muciniphila* ↑ *Odoribacter*, *Alistipes*, *Dehalobacterium*, *Defluviitalae*, *Oscillospira*, *Parabacteroides ↓*	SCFA: acetate, propionate, butyrate, valeric acid ↑	(Su et al., 2020)
A Purified Anthraquinone-Glycoside Preparatio	T2D	Physical barrier: ZO-1, occludin *↑*	*Lactobacillus, Roseburia, Akkermansia* ↑ *Desulfovibrio ↓*	SCFA: butyrate ↑	(Cui et al., 2019)
Hydroxysafflor yellow A	Obesity	Physical barrier: ZO-1, villus height, crypt depth *↑* Chemical barrier: colonic goblet cells/crypt ↑	*Akkermansia, Butyricimonas, Alloprevotella, Romboutsia ↑* *Lachnospiraceae ↓* *Bacteroidetes*/*Firmicutes* ratio↑	SCFA: acetate, propionate, butyrate ↑	(Liu et al., 2018a)
Vaccarin	T2D	Physical barrier: Intestinal alkaline phosphatase ↑, Claudin-1, occludin, ZO-1 ↑ Chemical barrier: goblet cells ↑	*Rikenellaceae*, *Bacteroides*, *Muribaculaceae ↑* *Lactobacillus*, *Lachnospiraceae*, *Desulfovibrio* ↓	SCFA: acetate, propionate, butyrate ↑	(Sun et al., 2021a)
Ginsenoside Rk3	Colonic inflammation	Physical barrier: ZO-1, occludin, claudin-1 ↑	*Bacteroides*, *Alloprevotella Blautia ↑*	SCFA: acetate, propionate, butyrate ↑	(Bai et al., 2021)
Diammonium Glycyrrhizinate	NAFLD	Physical barrier: ZO-1, occludin, claudin-1 ↑ Chemical barrier: goblet cells, mucin 2 ↑	*Proteobacteria*, *Deferribacteres*, *Bacteroidetes*, *Lactobacillus* ↑ *Firmicutes*, *Alistipes*, *Anaerotruncu*, *Desulfovibrio* ↓	SCFA: acetate, propionate, butyrate, isobutyrate, isovalerate, valerate ↑	(Li et al., 2018)
Berberine	Metabolic disorder	Physical barrier: ZO-1, occludin ↑ Chemical barrier: reduce the expression of mucin 2 to reduce the apoptosis of goblet cells	*Bacteroides*, *Escherichia-Shigella*, *Bifidobacterium*, *A.muciniphila*, *Butyricimonas*, *Coprococcus*, *Ruminococcus*, *Roseburia*, *phylum Firmicutes*, *phylum Bacteroidetes*, *C. scindens*, *C. hylemonae ↑* the propotion of *Bacteroidetes* and *Firmicutes ↑* *Vibrio desulfuricus*, *Enterobacter*, *C. hiranosis ↓*	SCFA: SCFA-producing bacteria ↑ BA: BAs-decomposing bacteria ↑	(Wang et al., 2022b)
Baicalin	Ulcerative intestinal lesions	Physical barrier: ZO-1, occludin, cingulin *↑*	*Roseburia*, *Ruminococcaceae UCG-*009, *Ruminococcaceae UCG-*010, *Ruminococcaceae UCG-*014, *Bifidobacterium*, *Akkermansia*, *Allobaculum*, *Ruminococcus* 2 ↑	SCFA: acetic acid, propionic acid, butyric acid, isobutyric acid, valeric acid, isovaleric acid ↑	(Wu et al., 2019a)
Salidroside	Obesity	Physical barrier: ZO-1, occludin *↑* Chemical barrier: mucin 2 *↑*	Phylum level: *Verrucomicrobiota* and *Actinobacteria ↑* Genus level: *Bacteroides*, *Actinobacteria*, *Parabacteroides*, *Dubosiella, Lactobacillus*, and *Bifidobacterium ↑* *norank_f_Muribaculaceae*, *Helicobacter*, *Ruminococcus_torques_group ↓*	SCFA: acetic acid, butyric acid, valeric acid, isobutyric acid, isovaleric acid, isohexanoic acid ↑	(Liu et al., 2023)
Ferulic acid	Obesity	Physical barrier: ZO-1, occludin, claudin-1 ↑	Phylum level: *Firmicutes ↑*, *Bacteroidetes ↓* Genus level: *Olsenella*, *Eisenbergiella*, *Dubosiella*, *Clostridiales_unclassified*, and *Faecalibaculum ↑*, *Lachnospiraceae_unclassified*, *Firmicutes_unclassified*, *Lachnospiraceae_NK4A136_group*, *Ruminiclostridium_9, Bacteroides*, and *Helicobacter ↓*	SCFA: acetate, butyrate, valerate ↑	(Tian et al., 2022)
Phlorizin	Obesity	Physical barrier: ZO-1, occludin, claudin-1 ↑ Chemical barrier: goblet cells ↑, mucus layer thickness ↑	*Bacteroidetes (*Phylum level*) ↑* *Clostridia*, *Bacteroidia* (Class level) *↑* *Desulfovibrionales*, *Bacteroidales* (Order level) *↑* *Desulfovibrion*, *Muribaculaceae (*Family *level) ↑* *Bilophila*, *GCA-900066575* (Genus level) *↑* *Firmicutes (*Phylum level*) ↓* *Mucispirilu Bilophila ↓*	SCFA: acetic acid, propionic acid, butyric acid ↑	(Zhang et al., 2020b)
2,3,5,4’-Tetrahydroxystilbene-2-O-*β*-D-glucoside	Acute colitis	Physical barrier: ZO-1, occludin ↑ Chemical barrier: goblet cells ↑	the ratio of *Firmicutes/Bacteroidetes* ↓ *Lachnospiraceae_NK4A136 ↑* *Proteobacteria*, *Helicobacter*, *Bacteroides*, *Parabacteroides* ↓	–	(He et al., 2021)

In “Improved barrier and corresponding altered indicators” and “Variation of gut microbial metabolites” parts, ↑ means the expressions of indicators or metabolites took on an increased trend, and ↓ means the expressions of those took on a decreased trend.

In “Variation of gut microbiota” part, ↑ means the abundance of the mentioned gut microbiota took on an increased trend, and ↓ means the abundance of those took on a decreased trend.

### 5.1 T2D

T2D is a prevalent chronic clinical condition characterized by absolute or relative inadequate insulin synthesis, often accompanied by IR and reduced sensitivity of target organs to insulin. T2D is associated with metabolic disorders involving fat, water, electrolytes, and other imbalances (Canfora et al., 2019; Yoo et al., 2020) The pathogenesis of T2D is closely linked to dysbiosis of the gut microbiota, obesity, genetic factors, and dysfunction of pancreatic islets (Ma et al., 2019). Growing evidence has supported the correlation between T2D occurrence and an imbalanced gut microbiota ecology, as well as damaged gut barrier (Cani et al., 2012). The density of *Faecalibacterium*, *Roseburia*, *Dialister*, *Flavonifractor*, *Alistipes*, *Haemophilus*, and *Akkermansia muciniphila* has shown a declining trend in the pathogenesis of T2D (Letchumanan et al., 2022). *Akkermansia muciniphila* plays a protective role in maintaining the gut chemical barrier and promoting glucose tolerance. Its decreased concentration has been associated with increased permeability of the intestinal tract (Letchumanan et al., 2022). Furthermore, T2D patients exhibit lower levels of SCFAs such as acetic acid, propionic acid, and butyric acid (Salamone et al., 2022). The dysbiosis of the gut microbiota community, along with the shortage of SCFAs, compromise the integrity and function of the gut barrier, thereby exacerbating T2D symptoms. In T2D patients, the physical barrier and microbial barrier of the gut are primarily affected (Ma et al., 2019). Notably, intestinal bacteria have been detected in the bloodstream of T2D patients, indicating evident damage to the gut barriers (Sircana et al., 2018). This damage facilitates the colonization of pathogenic microorganisms in the intestinal mucus layer and subsequent translocation of gut microbiota. As a result, endogenous infections and metabolic endotoxemia may occur (Ma et al., 2019).

Increased gut permeability and translocation of intestinal LPS are closely associated with metabolic inflammation in T2D. The compound of Scutellaria baicalensis and Coptis chinensis (SC) has been found to ameliorate these phenomena in T2D by modulating the gut microbiota and reinforcing the physical barrier in diabetic rats (Zhang et al., 2020a). Administration of SC led to a decline in the levels of *Proteobacteria*, *Enterobacteriaceae*, *Enterococcus*, *Escherichia-Shigella*, and *Enterobacter*, while promoting the abundance of certain bacteria belonging to *Lachnospiraceae* and *Prevotellacae*. Additionally, SC promoted the levels of TJ proteins (ZO-1, occludin, and claudin-1), which play a role in inhibiting intestinal inflammatory responses related to TLR-4/TRIF and TNFR-1/NF-*κ*B signaling pathways, thus preventing LPS translocation (Zhang et al., 2020a). Ultimately, these changes attenuated abnormal glucose metabolism and chronic inflammation caused by LPS leakage in T2D (Cani et al., 2012). Apart from reinforcing gut barriers, SCFAs elevated by SC can exert a beneficial effect on T2D by interacting with GPR43, which promotes the levels of GLP-1 (Zhu and Goodarzi, 2020). GLP-1 can improve insulin release, restore IR, inhibit glucagon secretion and promote satiety (Tolhurst et al., 2012).

In *db/db* mice (obese and diabetic), Andrographolide has demonstrated its potential to modulate the gut microbiota and improve metabolic health. Specifically, it can elevate the *Bacteroidetes/Firmicutes* ratio and promote the abundance of *Akkermansia muciniphila*, a bacterium known for its beneficial effects in delaying the development of diet-induced obesity and insulin resistance (Cani and de Vos, 2017). It also reversed the decreased SCFAs in mice by increasing the levels of acetate, propionate, butyrate, and valeric acid. Moreover, Andrographolide promoted the levels of mucin 2, enhancing the gut chemical barrier, as well as ZO-1 and occludin, enhancing the gut physical barrier. These effects may be associated with the downregulation of pro-inflammatory markers such as IL-6 and TNF-*α* after Andrographolide administration. Overall, based on these observed changes, Andrographolide has demonstrated its effectiveness in reducing oxidative stress and inflammation, hence alleviating glucose intolerance and insulin resistance (Su et al., 2020).

Ramulus Mori Alkaloids (SZ-A) have demonstrated therapeutic effects on inflammatory damage in T2D KKAy mice (Liu et al., 2021). SZ-A increased the concentration of propionate and acetate while significantly elevating the expression of MCT1 and SMCT1 (SLC5A8), which are crucial for the transcellular transfer of SCFAs into the intestinal epithelium. This resulted in enhanced consumption of SCFAs by intestinal cells (Liu et al., 2021). SZ-A also reduced the abundance of *Desulfovibrio*, *Alistipes*, and *Aerococcus*, while promoting those of *Bacteroides*, *Faecalibaculum*, and *Allobaculum*, thereby assisting in the restoration of the gut microbial barrier. Moreover, SZ-A enhanced the gut immune barrier by reducing the percentage of macrophages (CD11c+/F4/80+) and the cell mass of CD11c-positive monocytes (Liu et al., 2021). These effects worked together to downregulate the expression of inflammatory cytokines (IL-1*β*, IL-6, CCL4, CCL5) in the intestinal tract, thereby alleviating T2D (Liu et al., 2021).

In conclusion, TCM plays a role in alleviating T2D and its related symptoms by increasing the expression of TJ proteins, reinforcing the function of the gut physical barrier to attenuate LPS leakage. TCM also maintains the normal secretion of GLP-1 through the modulation of the gut microbiota community. Furthermore, TCM ensures an adequate supply of SCFAs and the normal function of SCFA transporters and receptors, which are essential for the normal function of gut barriers. Additionally, TCM regulates the gut immune barrier and reduces the levels of pro-inflammatory cytokines. Most importantly, most of these therapeutic functions are based on the regulation of the gut microbiota.

### 5.2 NAFLD and NASH

NAFLD encompasses a spectrum of diseases, ranging from simple steatosis to advanced NASH, and ultimately hepatocellular carcinoma. The pathogenesis of NAFLD is primarily driven by lipid accumulation, which leads to lipotoxicity and mitochondrial dysfunction. These alterations can trigger hepatocyte injury, inflammation, and varying degrees of fibrosis (Pierantonelli and Svegliati-Baroni, 2019). Inflammatory response is a hallmark of NASH pathogenesis, involving the release of various proinflammatory cytokines and hepatocyte damage (Lan et al., 2021). Apart from that, the activation of TLR hepatic inflammatory factors mediated by LPS and oxidative stress are crucial factors in the occurrence and development of NASH (Kiziltas, 2016). Importantly, a compromised gut physical barrier has been observed in NAFLD patients (Dai and Wang, 2015). Furthermore, under conditions of a high-fat diet, NAFLD individuals showed aberrant bacterial growth and altered microbiota composition when compared to healthy controls. Recent research has underscored the growing recognition of the significant role that gut barrier malfunction and the subsequent translocation of harmful bacteria and pathogen-associated molecular patterns (such as LPS) play in the development of hepatic inflammation and the progression of both NAFLD and NASH (Turner, 2009; Albillos et al., 2020; Ghosh et al., 2021).

BAs are an important factor related to the amelioration of NAFLD (Volynets et al., 2010). The levels of both primary and secondary BAs are observed to increase in NAFLD patients (Jiao et al., 2018). BAs not only suppress hepatic gluconeogenesis, facilitate insulin secretion and glycogen synthesis, and relieve inflammation, but also regulate the balance between Th17 and Treg cells to maintain the function of the gut immune barrier and alleviate inflammation (Shapiro et al., 2018). SCFAs help maintain the integrity of the gut physical barrier, preventing the migration of toxic compounds to the liver (Kelly et al., 2015; Wang et al., 2021). Butyrate, in particular, has shown the capability to inhibit inflammation in mice by promoting the differentiation of colonic anti-inflammatory regulatory T cells, thereby delaying the progression of NAFLD (Canfora et al., 2019).

The gut microbiota and its metabolites play a vital role in the development of NASH through their interaction with the liver, a relationship known as the gut-liver axis (Marra and Svegliati-Baroni, 2018; Albillos et al., 2020). TCM exhibits a comprehensive function in maintaining the gut-liver axis and alleviating NASH symptoms. For example, Fufang Zhenzhu Tiaozhi Capsule has been shown to attenuate NASH and progressive liver fibrosis in NAFLD mice by inhibiting hepatic inflammation, stellate cell activation, and collagen deposition. This is achieved by regulating tight junctions, consolidating intestinal barrier function, and restoring the dysregulated gut microbiota (*Bacteroidia*, *Verrucomicrobiae*, *Clostridia*, *Epsilonproteobacteria*) and its metabolites, especially BAs (Lan et al., 2022). Notably, the pool size, composition as well as chemical and signaling properties of BAs all are modulated by gut microbiota through enzymatic reactions such as deconjugation and epimerization (Ridlon et al., 2014; Parséus et al., 2017).

Another example is PTH, a TCM that has been demonstrated to protect the liver by lowering steatosis and inhibiting inflammatory cells (Zeng et al., 2024). Its administration in mice reversed diet-induced steatosis and liver injury by increasing the abundance of beneficial bacteria such as *Lactobacillus acidophilus* (*L. acidophilus*), *Lactobacillus plantarum*, *Lactococcus lactis*, *Bacillus subtilis* and *L. acidophilus* (Zeng et al., 2024), hence increasing the levels of therapeutic BAs, such as tauroursodeoxycholic acid (TUDCA), taurocholic acid (TCA), taurodeoxycholic acid (TDCA), and glycocholic acid (GCA). Furthermore, PTH treatment lifted the expression of E-cadherin, thereby enhancing the integrity of gut physical barrier (Zeng et al., 2024).

Besides, Zhishi daozhi decoction (ZDD) effectively attenuated uncontrolled inflammatory responses in high-fat diet-induced NAFLD mice by optimizing the gut microbial structure, increasing the levels of *Faecalibacterium* and *Bacteroidetes*, and reducing those of *Brautia* and *Colidextribacter* (Bi et al., 2023). Additionally, ZDD increased the concentration of acetic acid, butyric acid, and propionic acid, as well as the levels of ZO-1 and occludin, thereby strengthening the gut physical barrier and promoting NAFLD recovery (Bi et al., 2023). And in treatment of NAFLD rats, the opportunistic pathogen *Escherichia/Shigella* was decreased and the SCFA producer *Collinsella* was increased by Qushi Huayu Decoction. Its active components can also improve Treg cells activity. These effects contributed to repairing gut immune barrier and then inhibiting the exposure of liver to harmful substances such as LPS and other noxious gut microbiota products (Feng et al., 2017).

In conclusion, damaged gut barriers are commonly observed in NAFLD and NASH hosts. BAs and SCFAs play crucial roles in the therapeutic functions of NAFLD. TCM can restore the dysregulated gut microbiota community and simultaneously restore levels of corresponding therapeutic gut microbiota metabolites in the treatment of NAFLD. Based on the regulation of gut microbiota and its metabolites, multiple gut barriers can be repaired or enhanced to prevent the progression of NAFLD or NASH.

### 5.3 UC

UC is a chronic inflammatory bowel disease prone to relapsing and characterized by clinical symptoms such as diarrhea, weight loss, abdominal pain, and bloody stool. It is also associated with an increased risk of colorectal cancer. The lesions in UC are typically found in the colonic mucosa and submucosa (Ungaro et al., 2017; Dong et al., 2020). While the exact etiology and pathogenesis of UC are not fully understood, studies have shown that it is mainly influenced by factors such as predisposition, dysfunction of the intestinal epithelial barrier, dysbiosis of the gut microbiota, immune response disorders, and environmental factors (Zhang and Li, 2014).

Apart from inflammation-induced injuries in the gastrointestinal tract, patients with UC also suffer from mucosal inflammation extending from the colon to the rectum and gut barrier dysfunction, which is principally caused by depleted colonic goblet cells and increasing permeability of the gut chemical barrier (Johansson et al., 2014).The pathogenesis of UC involves the participation of innate lymphoid cells (Buonocore et al., 2010) and cellular immunity, in which ILC3, T-helper-2 (Th2), and IL-4, IL-1*β*, IL-13 TNF-*α* are involved (Ungaro et al., 2017; Miao et al., 2021). Furthermore, a decreased biodiversity of the gut microbiota has been associated with the development of UC. Specifically, UC patients have been found to have decreased levels of *Firmicutes* and increased those of *Gamma-proteobacteria* and *Enterobacteriaceae* (Frank et al., 2007).

TCM has shown promise in the treatment of intestinal tract injuries in UC. For example, Baitouweng decoction (BTW), a commonly used formula in UC therapy, has been found to increase the abundance of beneficial bacteria such as *Lactobacillus* and *Akkermansia*, while decreasing the abundance of harmful bacteria such as *Escherichia-Shigella* in UC mice (Chen et al., 2021b). BTW also increased the production of butyrate, propionate and acetate and enhanced the expression of ZO-1 and occludin (Miao et al., 2021). Additionally, BTW can modulate the Th17/Treg cell balance, thereby preventing inflammatory cytokines like TNF-*α* and IL-1*β* from damaging TJ proteins and further compromising intestinal integrity (Miao et al., 2021). These changes helped strengthen both the physical and immune barriers of the gut, leading to improvements in inflammatory symptoms associated with UC.

Another TCM remedy, the aqueous extract of Paeoniae radix alba ((paeonia lactiflora pall) (PRADG)), has also shown to protect the intestinal tract against inflammation. PRADG attenuated the population of Th17 cells and downregulated the IL-23/IL-17 axis, thereby reducing inflammation. Moreover, PRADG promoted the restoration of injured goblet cells, epithelial cells, and colonic crypts in mice with DSS-induced colitis. This restoration enhanced both the physical and chemical barriers of the gut (Yan et al., 2022), preventing detrimental effects caused by local/systematic inflammatory or immunology responses when gut bacterial metabolites translocation occurring (Yan et al., 2022). And after PRADG treatment, the abundance of *Bacteroidetes*, *Verrucomicrobia*, *Actinobacteria*, *norank_f_Muribaculaceae*, *Lactobacillus*, and *Akkermansia* were increased and those of *Proteobacteria*, *Fusobacteria*, *Bacteroides*, *Escherichia-Shigella*, *Romboutsia*, and *Fusobacterium* were decreased.

Additionally, Ginsenoside Rg 1 can restore the colonic injury and colonic inflammation via reversing general gut microbiota structure and abundance disturbed in DSS-induced mice (Cheng et al., 2022b) and promoting the relative abundance of beneficial bacteria such as *Lactobacillus Akkermansia norank_f_Muribaculaceae*, which can catabolize Trp into its metabolites by activating AhR agonist (Cheng et al., 2022b). These gut microbiota metabolites and AhR agonists, including (3-indolylacrylic acid (ILA), indole propionic acid (IPA), IALD, nicotinamide (Nam)), decrease the levels of pro-inflammatory cytokines, thereby protecting the physical barrier of the gut. Moreover, the interface between AhR and its ligands (ILA, IPA, IALD) can protect this barrier by increasing the production of anti-inflammatory cytokines such as IL-22 (Cheng et al., 2022b).

In conclusion, TCM can treat UC via modulation of physical barrier, chemical barrier, immunological barrier, and the mechanisms are linked to modulation of gut microbiota and its metabolites.

## 6 Conclusion and perspectives

The gut barrier plays a crucial role in maintaining the normal function and balance of the intestinal tract. In this review, we provide an overview of the composition and function of the four main gut barriers. Additionally, we analyze the intricate interactions between the predominant gut microbiota, its metabolites, and TCM compounds. These interactions between TCM compounds and the gut microbiota hold great potential for therapeutic applications. Understanding the mechanisms involved in these interactions will not only enhance our knowledge of TCM pharmacodynamics but also pave the way for the development of novel therapeutics targeting gut microbiota imbalance and impaired gut barrier. Although great progresses have been made in understanding how TCM can improve gut microbiota, there are still considerations that warrant attention in future studies examining the mechanisms by which TCM can enhance gut barrier function and treat associated diseases.

The complexity of TCM compounds and the diversity of gut microbiota present significant challenges in studying the pharmacodynamics of TCM. Factors such as different origins, processing methods, and combinations can affect the therapeutic properties and functions of TCM when administered clinically. Therefore, it is crucial to establish standardized and processed criteria to ensure consistent and effective therapeutic outcomes.

In order to demonstrate the causal relationship between TCM and its medicinal effects, it is important to refine the measurement of TCM effects on the microbiota and assess gut barrier indicators in a more comprehensive manner. Merely testing the composition or ratio of gut microbiota, along with a few simple biochemical and immunological indicators, may not provide sufficient evidence. Additionally, there is a lack of in-depth exploration of the pathways of action, related enzymatic pathways, and therapeutic outcomes interpretated by TCM theory.

To better understand and regulate the gut microbiota response to TCM, detailed analysis of intestinal contents is necessary. This will help identify specific changes and enable personalized medication approaches. Exploring the molecular mechanisms and signal pathways involved in the interaction between TCM and the gut microbiota is also important for achieving desired therapeutic outcomes. Moreover, while there have been studies investigating the metabolism of individual active ingredients (monomers) of TCM by the gut microbiota, research on drug interactions becomes more challenging when multiple components are metabolized simultaneously. Therefore, future studies should focus on analyzing the impact of the gut microbiota on the metabolism of TCM as a whole. This comprehensive approach will enhance our understanding and utilization of TCM in clinical practice.

## Author contributions

YZ: Investigation, Methodology, Visualization, Writing – original draft, Writing – review & editing. JL: Writing – review & editing, Funding acquisition, Investigation. DZ: Writing – review & editing, Investigation. HC: Investigation, Writing – review & editing. JW: Investigation, Writing – review & editing. WF: Conceptualization, Funding acquisition, Supervision, Writing – review & editing, Writing – original draft. CP: Conceptualization, Funding acquisition, Supervision, Writing – review & editing.
